# Toxic Tau Oligomers Modulated by Novel Curcumin Derivatives

**DOI:** 10.1038/s41598-019-55419-w

**Published:** 2019-12-12

**Authors:** Filippa Lo Cascio, Nicha Puangmalai, Anna Ellsworth, Fabio Bucchieri, Andrea Pace, Antonio Palumbo Piccionello, Rakez Kayed

**Affiliations:** 1 Mitchell Center for Neurodegenerative Diseases, University of Texas Medical Branch, Galveston, TX 77555 USA; 2 Departments of Neurology, Neuroscience and Cell Biology, University of Texas Medical Branch, Galveston, TX 77555 USA; 3 Department of Biomedicine, Neurosciences and Advanced Diagnostic (BiND), University of Palermo, Palermo, 90127 Italy; 4 Department of Biological, Chemical and Pharmaceutical Sciences and Technologies - STEBICEF, University of Palermo, Palermo, 90128 Italy

**Keywords:** Biochemistry, Biophysics, Cell biology, Chemical biology, Drug discovery, Neuroscience

## Abstract

The pathological aggregation and accumulation of tau, a microtubule-associated protein, is a common feature amongst more than 18 different neurodegenerative diseases that are collectively known as tauopathies. Recently, it has been demonstrated that the soluble and hydrophobic tau oligomers are highly toxic *in vitro* due to their capacity towards seeding tau misfolding, thereby propagating the tau pathology seen across different neurodegenerative diseases. Modulating the aggregation state of tau oligomers through the use of small molecules could be a useful therapeutic strategy to target their toxicity, regardless of other factors involved in their formation. In this study, we screened and tested a small library of newly synthesized curcumin derivatives against preformed recombinant tau oligomers. Our results show that the curcumin derivatives affect and modulate the tau oligomer aggregation pathways, converting to a more aggregated non-toxic state as assessed in the human neuroblastoma SH-SY5Y cell line and primary cortical neuron cultures. These results provide insight into tau aggregation and may become a basis for the discovery of new therapeutic agents, as well as advance the diagnostic field for the detection of toxic tau oligomers.

## Introduction

Millions of people worldwide are affected by age-related tauopathies, including Alzheimer’s disease (AD), which are characterized by the pathological accumulation of tau aggregates^1–3^. Tau is a natively unfolded protein involved in microtubule stabilization and axonal transport. However, under pathological conditions, tau detaches from the microtubules, causing their instability and disassembly. As a consequence of this detachment, there is a perturbation of cytoskeletal stability and axonal transport^4,5^. Unbound tau can self-aggregate to form soluble oligomers (TauO) that combine into paired helical filaments (PHFs)^6–8^. The PHFs then aggregate into large insoluble fibrils, known as neurofibrillary tangles (NFTs)^9^. Although NFTs have been assumed to be the main pathological hallmark in tauopathies, recent studies have demonstrated that the accumulation of the smaller, soluble and dynamic tau oligomers, as well as neuronal loss, precede the formation of NFTs and the clinical manifestation of AD symptomatology^10,11^. These evidences suggest that tau oligomers are the toxic entities playing a crucial role for disease onset and acting as an efficient seed in the misfolding and the propagation of the pathology^12,13^. Moreover, this evidence supports tau as being a potential target for the development of successful disease-modifying therapeutics^1,14^. Unfortunately, the aggregation and subsequent internalization of misfolded proteins by cells, and their spreading into neighboring or anatomically connected cells, contributes to what makes the treatment of tauopathies difficult^15,16^. This phenomena highlights the need to diagnose these disorders earlier and more effectively to begin treatment prior to the initiation and spread of the pathology^17–19^. Despite tau being an intracellularly expressed protein, increasing evidence suggests the presence of extracellular tau aggregates and their role in tau propagation. Thus, it has been proposed that extracellular treatments may be as equally important as intracellular treatments in disease prevention^20–25^. In recent years, tau aggregation inhibitors have been a focus of great interest as potential disease-modifying drugs. The search for non-toxic tau aggregation inhibitors capable of easily crossing the Brain-Blood-Barrier (BBB) have led to the discovery of several synthetic, as well as naturally occurring, small molecules able to inhibit amyloidogenic protein aggregation^23,26–29^. Curcumin, which is a polyphenol extracted from the plant *Curcuma longa*, has been shown to play an important role in the prevention and treatment of many diseases, including neurodegenerative disorders^30–33^.It has been demonstrated that curcumin alters the misfolding of many amyloid proteins through the disruption of π-stacking due to the presence of conjugated phenol residues^34,35^. Indeed, it significantly reduces amyloid beta (Aβ) and tau pathology in transgenic AD mouse models^35–37^. Curcumin additionally labels amyloid deposits both *ex vivo* and *in vivo* by disrupting existing plaques and partially restoring distorted neurites in transgenic AD mice^38^. In addition, curcumin decreases levels of hyperphosphorylated tau in cells and mice by binding to fibrillar tau^39^. Recently, curcumin was also found to selectively suppress soluble tau dimers in aged Htau mice and to improve tau-mediated neuronal dysfunction and neuritic abnormalities in *C. Elegans*^37,40^. Hence, extensive preclinical studies have proposed curcumin as a potential therapeutic approach against AD and related neurodegenerative diseases, but no clinical trials have been successful^31,41^. Their failures may be due to curcumin’s poor solubility in aqueous buffers and low brain bioavailability following oral administration^31,42–46^. As a result, alternative formulations and drug delivery systems (e.g. liposomes, nanoparticles) have been formulated, as well as novel curcumin derivatives that have been synthesized to boost and improve its bioavailability^47–53^. Based on these previous studies, we decided to evaluate the effects of curcumin and novel curcumin derivatives on oligomeric tau species using our *in vitro* preparation of tau oligomers. In this study, we used *in vitro* approaches to investigate the potential neuroprotective properties of curcumin and newly synthesized curcumin-derived small molecules by converting the aggregation state of toxic tau oligomers to a non-toxic one, as assessed by cell-based assays.

## Results

### Curcumin effects on preformed toxic tau oligomers

**Figure 1:**
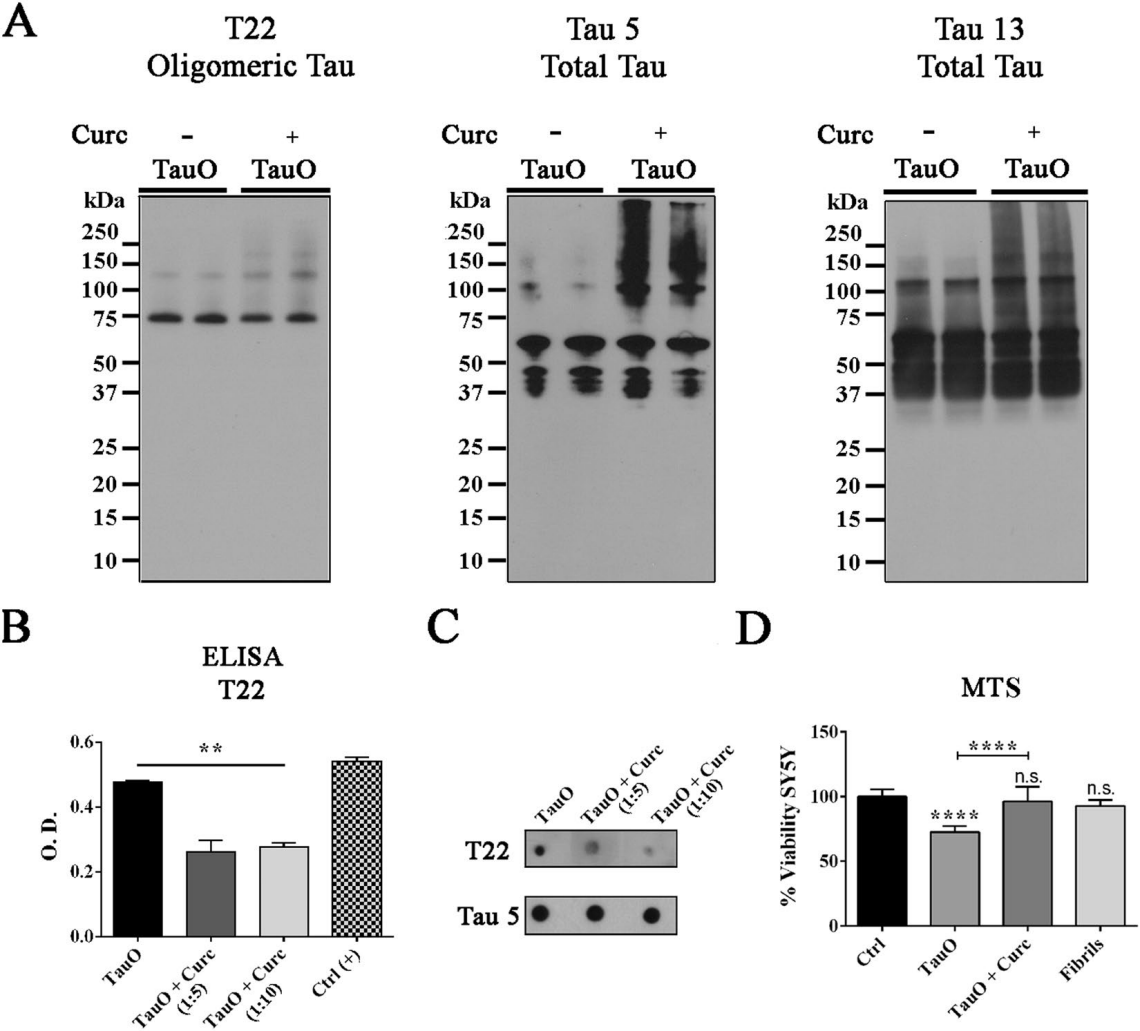
Biochemical and cytotoxicity analyses of oligomeric tau treated with curcumin and untreated control. (**A)** Western blot analyses of tau oligomers probed with the oligomeric tau antibody, T22 and generic total tau antibodies, Tau 5 and Tau 13. Curcumin interacts and alters the aggregation states of preformed TauO. **(B)** ELISA analysis of oligomeric tau treated with increased concentration of curcumin shows a significant decrease in T22 immunoreactivity as compared to the untreated TauO. **(C)** Dot blot analysis show decreased levels of oligomeric tau in the presence of curcumin. **(D)** Viability percentage of cultured SH-SY5Y human neuroblastoma cells exposed to 2 µM TauO or 2 µM TauO pre-incubated with curcumin and controls. SH-SY5Y cells given TauO pretreated with curcumin had significantly higher cells viability when compared to TauO alone and Ctrl. Data in B and D were compared by one-way analysis of variance (ANOVA) followed by Dunnett’s multiple comparison test: **p < 0.01, ***p < 0.001. Bars and error bars represent the mean and standard deviation.

We first evaluated the effect of curcumin using our *in vitro* preparation of TauO. Therefore, highly purified oligomeric tau species were incubated with and without curcumin (1:5 and 1:10 molar ratio) at room temperature on an orbital shaker, under oligomerization conditions. Tau oligomers in the absence and presence of curcumin were then biochemically evaluated using the oligomer-specific antibody, T22, and generic total tau antibodies, Tau 5 and Tau 13 (Fig. 1). Western blot analyses showed that curcumin interacts with tau oligomers by promoting the formation of higher molecular weight tau aggregates (Fig. 1A).

In addition, direct enzyme linked immunosorbent assay (ELISA) and dot blot analyses showed a significant decrease in oligomers, as seen by the decreased T22 immunoreactivity (Fig. 1B,C). Next, the toxicity of curcumin-induced aggregates was assessed by 3-(4, 5-dimethylthiazol-2-yl)-5-(3-carboxymethoxyphenyl)-2-(4-sulfophenyl)-2H-tetrazolium, inner salt (MTS) using the human neuroblastoma cell line, SH-SY5Y. Cells were exposed to untreated TauO or TauO in the presence of curcumin (final concentration 10 μM) for 24 hours. SH-SY5Y cell viability decreased significantly after treatment with TauO, while the treatment with curcumin rescued cells from TauO-induced toxicity, as seen by the higher cell viability compared to the cells exposed to untreated TauO (Fig. 1D). All together, these results indicate that curcumin has neuroprotective effects against toxic tau oligomers.

### Synthesis and screening of novel curcumin analogs

**Figure 2:**
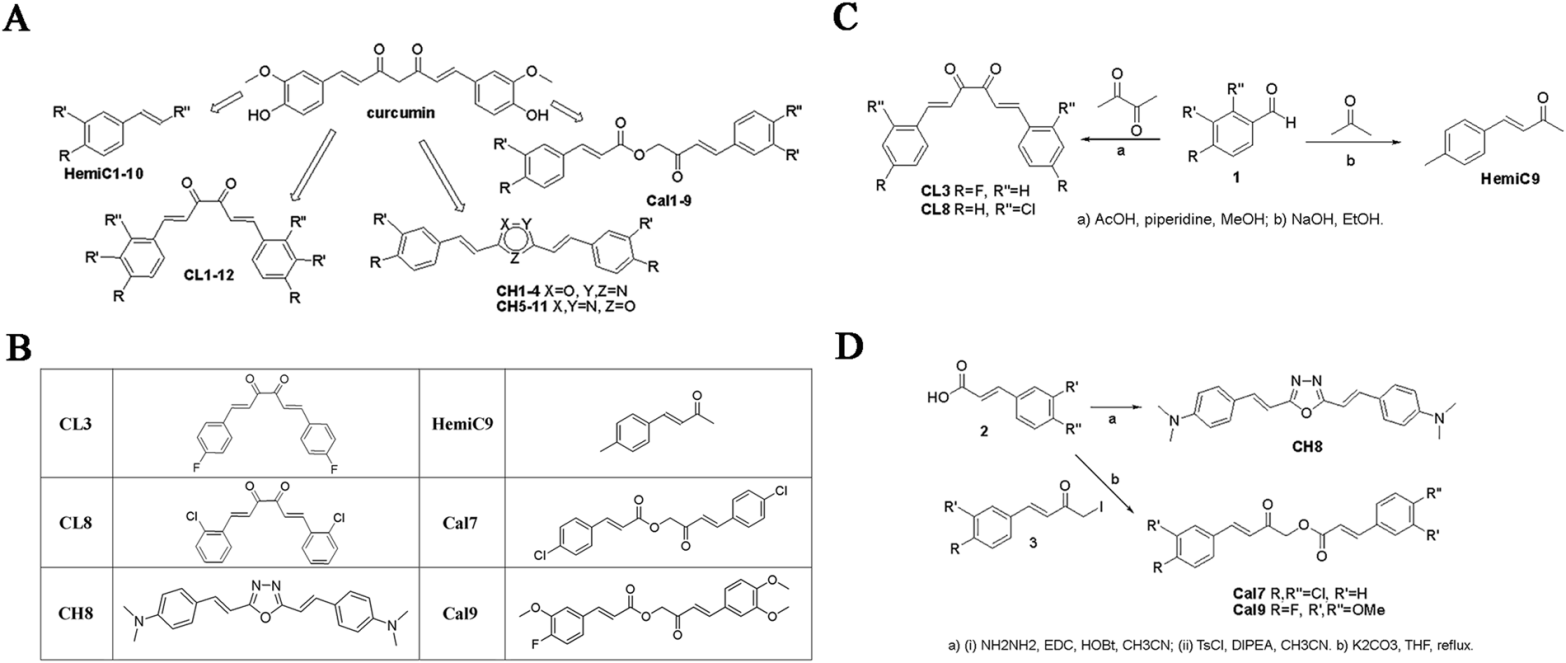
Structure of curcumin and newly synthesized curcumin derivatives. (**A**) The library of our curcumin derivatives consists of four different classes: Hemi-curcuminoids (**HemiC**); Curcumin-like (**CL**); Heterocyclic curcumin-like (**CH**) and Calebin-A derivatives (**Cal**). (**B**) Curcumin derivatives showing higher activity in modulating the aggregation of preformed tau oligomers and selected for additional *in vitro* testing. (**C**) Synthesis of compound **CL3**,**8** and **HemiC9**. (**D**) Synthesis of compound **CH8**, **Cal7,9**.

To overcome the poor solubility of curcumin in aqueous buffers and its low cerebral bioavailability, novel curcumin derivatives were synthesized. Our curcumin-derived library of small molecules is comprised of four different groups of compounds with the potential to interact and modulate the aggregation state of TauO such that the progression of tauopathy can be slowed; this is accomplished by neutralizing their toxicity and internalization potency (Fig. 2A). In particular, heterocyclic derivatives with oxadiazole nucleus and Calebin-A derivatives were also included in this study due to their ability to interact with Aβ amyloid^54–56^. The complete library and synthetic details of all synthesized compounds are reported on Supplementary Information (SI).

All our derivatives are characterized by the removal of the β-di keto moiety that is assumed to be responsible for the well-known shortcomings of curcumin^57^. The efficacy of the curcumin derivatives was tested *in vitro* by screening the entire small compound library by group directly against preformed recombinant TauO. Our biochemical screening shows that the curcumin-derived small molecules can directly interact and perturb the aggregation state of preformed TauO (Figs. S1–S4).

**Figure 3:**
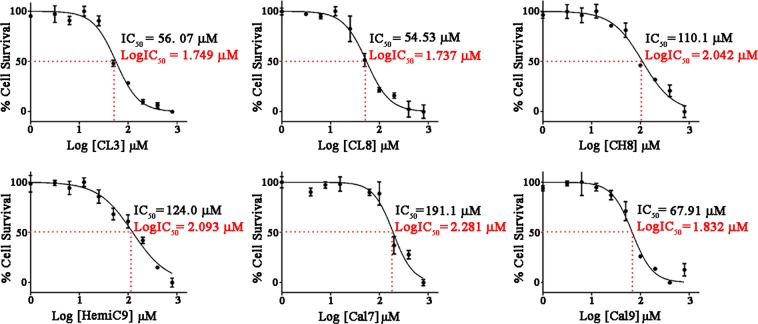
Curcumin derivatives effects on cell viability. Curcumin derivatives cytotoxicity on human neuroblastoma SH-SY5Y cell line was assessed by an MTS assay to determine the IC_50_ values for **CL3**, **CL8**, **CH8**, **HemiC9**, **Cal7** and **Cal9** following treatment with increasing concentration of the compounds (0–800 μM) for 24 hours. Values are presented as the mean ± SD (n = 3).

Six compounds were selected due to their higher activity in modulating toxic TauO and subjected to additional *in vitro* testing to validate their effects on TauO (Fig. 2B). Synthesis of these compounds was accomplished as reported in Fig. 2C,D. Compounds **CL3,8** were obtained through a double aldol condensation on the diacetyl of the appropriate aromatic aldehyde **1**^58^. Similarly, **HemiC9** was obtained by means of Claisen-Schmidt Aldol condensation of acetone under basic conditions (Fig. 2C)^59^. Heterocyclic derivative **CH8** was obtained in a two-step procedure from the corresponding cinnamic acid derivative **2** (Fig. 2D)^60^. Derivatives **2** were also employed for the synthesis of Calebin-A and its analogs **Cal7,9** through a substitution reaction on iodoketone **3** as shown in Fig. 2D. This method allows the obtainment of target products avoiding the use of protecting groups^61^. One of the criteria used to select these small molecules was evaluating their toxicity profile. The cytotoxicity of each curcumin derivative was examined by exposing the cultured human neuroblastoma SH-SY5Y cell line to a continuous range of small molecule concentrations (0–800 μM) and monitoring cell survival after 24 hours of treatment by an MTS assay. The dose-response curves show that the curcumin derivatives have a very low-toxicity profile with an IC_50_ ranging from 54.53 μM for **CL8** to 191.1 μM for **Cal7** (Fig. 3).

### Curcumin analogs promote tau oligomers aggregation

**Figure 4:**
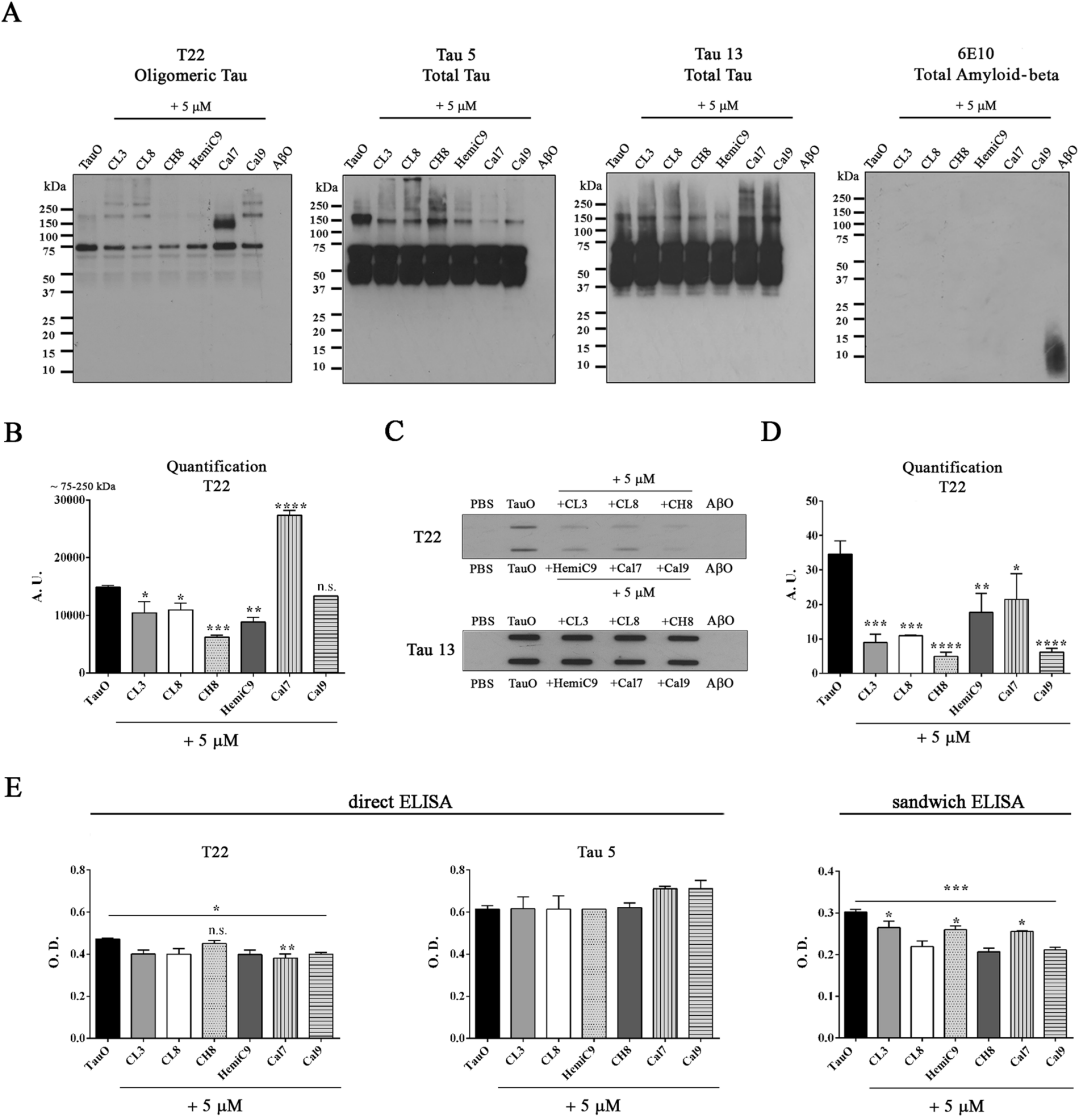
Biochemical analyses of oligomeric tau with and without curcumin derivatives. (**A)** Western blot of tau oligomers in the absence and presence of curcumin analogs (final concentration 5 μM) probed with the oligomeric tau antibody, T22 and total tau antibodies, Tau 5 and Tau 13 and the control anti-Aβ antibody, 6E10. (**B**) The incubation with the compounds modulates the aggregation states of preformed tau oligomers as seen by T22 quantification analysis. **(C,D)** Filter Trap assay of tau oligomers alone and pretreated with curcumin derivatives probed with T22 and Tau 5. Curcumin-derived small molecules alter the aggregation pathways of tau oligomers resulting in decreased T22 immunoreactivity as compared to the untreated TauO. **(E)** ELISA analyses shows that the selected compounds decrease tau oligomer levels as seen by the reduced T22 immunoreactivity and no significant changes in total tau protein as assessed by Tau 5 antibody. The reduction of tau oligomers was confirmed by sandwich ELISA, using T22 as capture antibody and Tau 5 as detection antibody. Data in B, D and E were compared by one-way analysis of variance (ANOVA) followed by Dunnett’s multiple comparison test: *p < 0.05; **p < 0.01; ***p < 0.001; ****p < 0.0001. Bars and error bars represent the mean and standard deviation.

TauO were further characterized *in vitro* in both the absence and presence of the selected curcumin derivatives to evaluate and compare their effects on TauO under the same conditions (Fig. 4). Indeed, oligomeric tau species were incubated with and without curcumin derivatives (final conc. 5 µM) and were evaluated biochemically using the anti-oligomeric tau antibody, T22, as well as generic tau antibodies, Tau 5 and Tau 13. Western blot analyses revealed that curcumin-derived small molecules interact with TauO, resulting in decreased oligomer levels or leading to the formation of higher molecular weight tau aggregates (Fig. 4A,B). Moreover, filter trap assay and direct ELISA confirmed that curcumin derivative interactions with TauO result in decreased oligomeric levels, as seen by the decreased T22 immunoreactivity, while no changes were observed in total tau levels as detected by Tau 5 immunoreactivity. In addition, a quantitative reduction of oligomeric tau, upon treatment with curcumin derivatives, was also evaluated by sandwich ELISA using T22 as capture antibody and Tau 5 as detection antibody (Fig. 4C–E).

### Biophysical characterization of curcumin derivatives-induced aggregates

**Figure 5:**
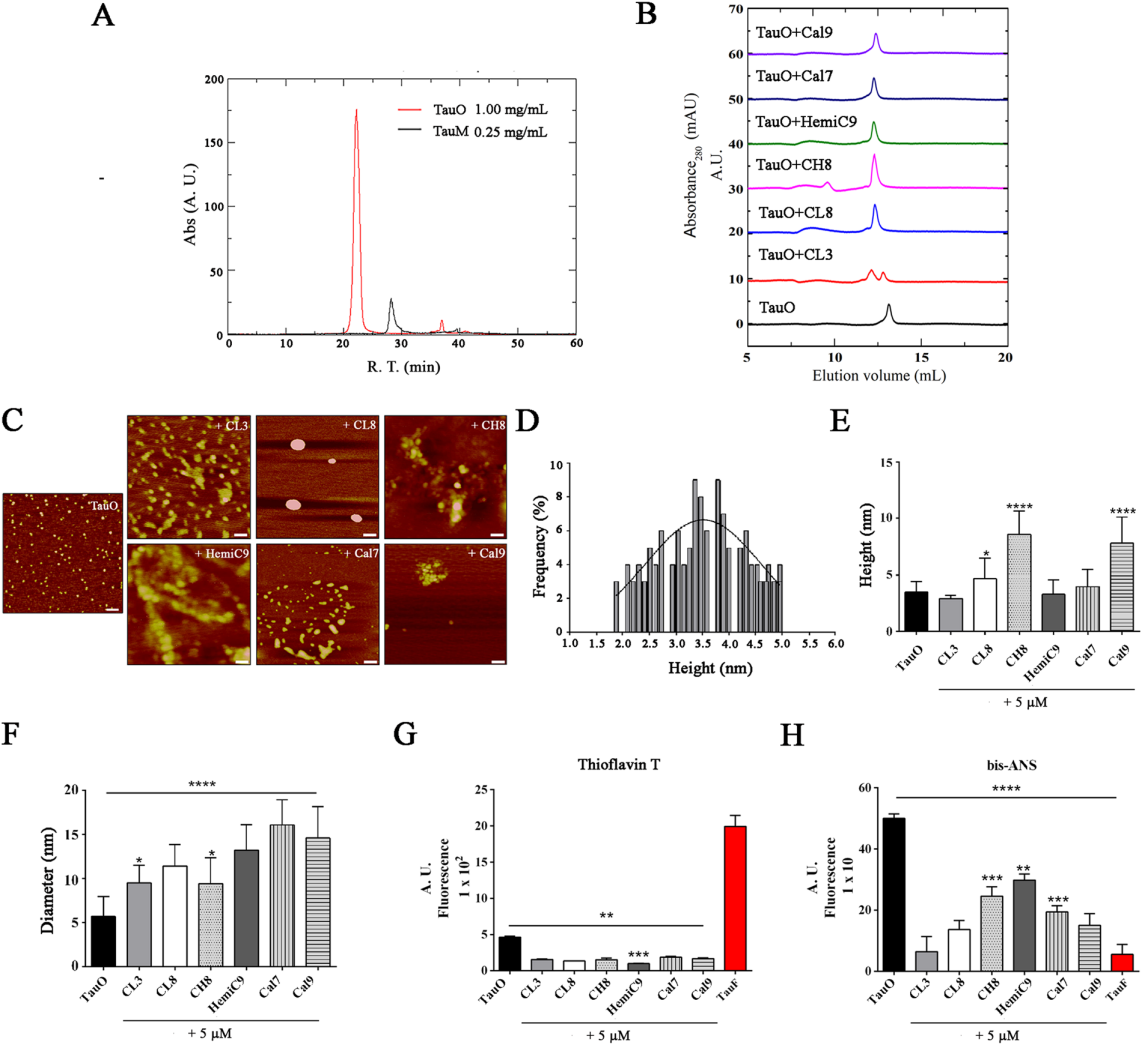
Biophysical characterization of tau oligomers with and without curcumin derivatives. (**A**) FPLC chromatogram of tau oligomers showing that the main peak is ~120–150 kDa (tau dimer/trimer). (**B**) SEC chromatograms of tau oligomers alone and in the presence of curcumin derivatives. (**C**) Atomic Force Microscopy images of TauO and TauO after treatment with 5 μM of curcumin derivatives for **CL3**, **CL8**, **CH8**, **HemiC9**, **Cal7** and **Cal9**; Scale bars = 100 nm. (**D)** Size distribution histogram of TauO shows that tau oligomers have an average height of 3.36 nm. (**E,F**) AFM analyses shows the ability of the compounds to modulate TauO aggregation states converting TauO into much larger aggregates as seen by modulation of tau aggregate heights (**E**) and sizes (**F**). (**G,H**)Tau oligomers alone or in the presence of 5 μM of curcumin derivatives were assessed by Thioflavin T and bis-ANS spectroscopy. Thioflavin T spectroscopy analysis (**G**) show no presence of fibrils formation after incubation with the compounds and preformed oligomers pretreated with curcumin analogs show reduced binding with bis-ANS as compared to untreated TauO (**H**). Data in (**E**–**H**) were compared by one-way analysis of variance (ANOVA) followed by Dunnett’s multiple comparison test: *p < 0.05; **p < 0.01; ***p < 0.001. Bars and error bars represent the mean and standard deviation.

In addition, we further investigated the effects of curcumin derivatives on tau oligomers by biophysically characterizing curcumin derivative-induced aggregates (Fig. 5). Fast protein liquid chromatography (FPLC) was used to purify tau oligomers, detecting a main peak at ~120–150 kDa (tau dimer/trimer) as seen in Fig. 5A. Moreover, to gain more insights into the structure and distribution of tau aggregates upon treatment with the curcumin derivatives, tau aggregates were analyzed by size-exclusion chromatography showing that the resultant tau aggregates have an higher molecular weight peaks as compared to the untreated tau oligomers (Fig. 5B). The morphology of tau oligomers was also evaluated by Atomic Force Microscopy (AFM) before and after treatment with the curcumin derivatives (Fig. 5C). AFM images of untreated tau oligomers displayed their classically homogeneous spherical morphology with the majority of the oligomers with a high of 3.36 nm as shown by the size distribution histogram (Fig. 5D). AFM analyses of TauO in the presence of the curcumin-derived small molecules show that preformed tau oligomers are converted into larger tau aggregates as seen in Fig. 5E,F. These data are consistent with Thioflavin T (ThT) and the 4,4′-dianilino-1,1′-binaphthyl-5,5′-disulfonic acid, dipotassium salt (bis-ANS) fluorescence assays that showed no fibril formation after incubation with curcumin derivatives and decreased binding of hydrophobic oligomers with bis-ANS, respectively, as shown in Fig. 5G,H. Moreover, both negative and positive controls were used, and readings were corrected for the background fluorescence to account for any intrinsic fluorescence of the compounds in the bis-ANS and ThT measurements. Taken together, these results suggest that curcumin derivatives decrease the levels of tau oligomers, affecting their morphology by promoting the formation of larger tau aggregates.

### Curcumin analogs rescue tau oligomers-associated neurotoxicity in neuronal cultures

**Figure 6:**
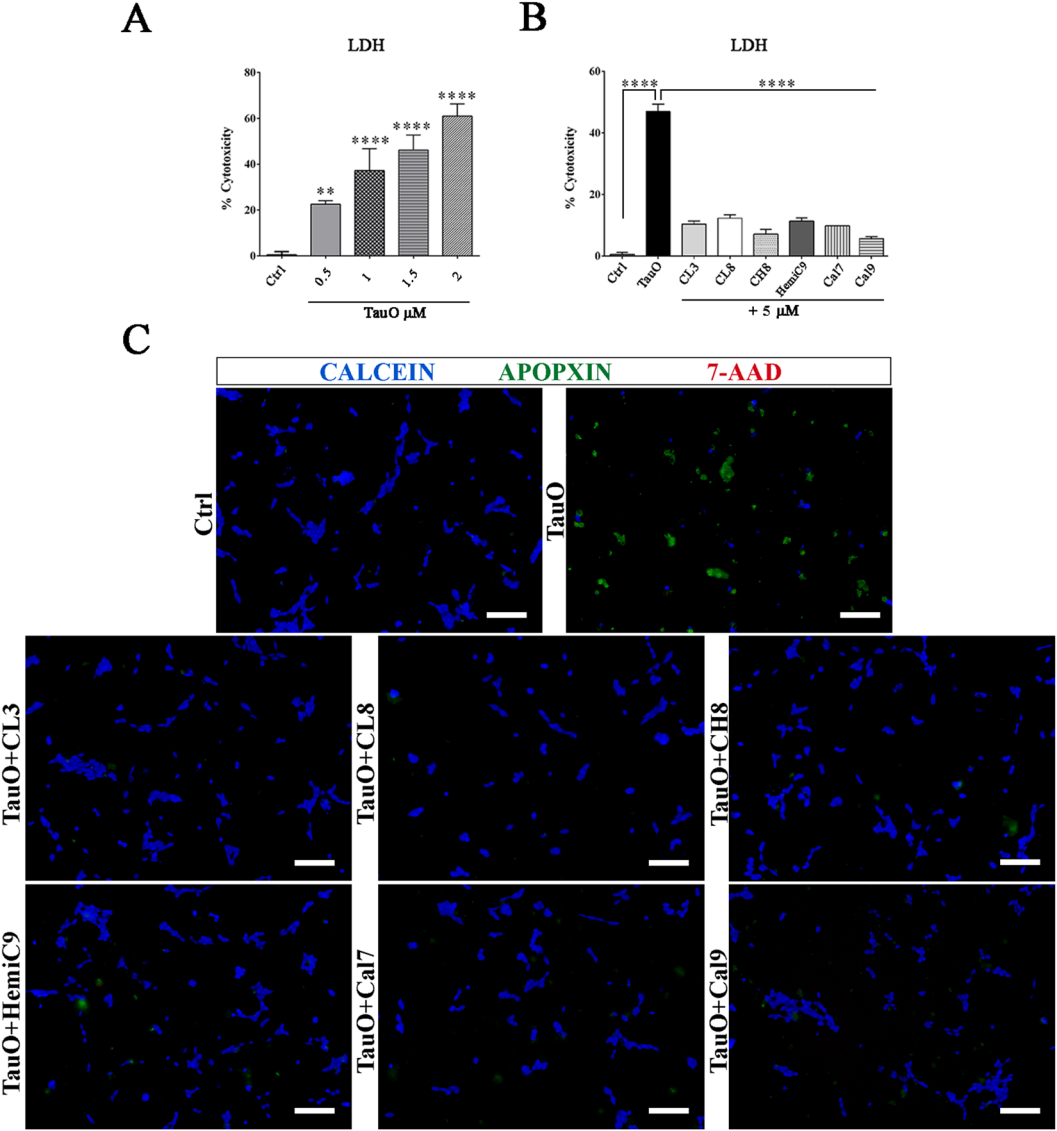
Curcumin derivatives-induced aggregates cytotoxicity in human neuroblastoma cell line. (**A**) Dose-dependent cytotoxicity of TauO in SH-SY5Y cells after 24 hours of treatment. (**B**) SH-SY5Y cells cytotoxicity after exposure to 2 µM TauO, or 2 µM TauO in the presence of curcumin derivatives (final concentration 5 µM) and untreated control (Ctrl). Cells exposed to TauO had significantly higher LDH release as compared to the untreated control or cells treated with TauO in the presence of the compounds. Each experiment was performed in triplicate (n = 3). (**C**) SH-SY5Y cells treated for 24 hours with 2 µM of TauO or TauO in the presence curcumin derivatives were incubated with Apopxin to detect apoptosis (green), 7-AAD for necrosis (red) and CytoCalcein Violet 450 to detect viable cells (blue) and imaged by immunofluorescence microscopy. Scale bar = 100 µm. Data in A and B were compared by one-way analysis of variance (ANOVA) followed by Dunnett’s multiple comparison test: **p < 0.01, ****p < 0.0001. Bars and error bars represent means and standard deviations performed.

Next, TauO-associated toxicity was assessed using the human neuroblastoma cell line, SH-SY5Y (Fig. 6). Cells were exposed for 24 hours to increasing concentrations of TauO (0.5–2 µM). The toxicity of TauO was evaluated in a lactate dehydrogenase (LDH)-based assay, showing a dose-dependent cytotoxicity of TauO, as the amount of LDH released from damaged cells is indicative of cellular cytotoxicity and cytolysis (Fig. 6A). Furthermore, we investigated the ability of curcumin derivatives to rescue cells from TauO-induced toxicity. SH-SY5Y cells were exposed to 2 µM TauO or TauO in the presence of curcumin derivatives. Interestingly, cells incubated with TauO in the presence of curcumin derivatives had a significantly lower LDH release as compared to those cells exposed to untreated TauO (Fig. 6B). In addition, the toxicity of TauO in the absence and presence of curcumin analogs was also evaluated by performing an apoptosis/necrosis assay in SH-SY5Y cells. The treatment was followed by fluorescence live cell imaging to monitor apoptotic, necrotic and viable cells combined simultaneously with bright field microscopy to better visualize and evaluate the details and morphology of viable cells in comparison to those undergoing apoptosis and cell death (Fig. 6C). An early event in apoptosis is the phosphatidylserine (PS) exposure from the inner to the outer side of the plasma membrane that was detected by staining with Apopxin, while necrotic and viable cells were stained with 7-Aminoactinomycin D (7-AAD) and CytoCalcein Violet 450, respectively. The PS translocation precedes the loss of membrane integrity which accompanies the later stages of cell death resulting from either apoptotic or necrotic processes. The fluorescence microscopy observations confirmed the previous toxicity screens. Indeed, SH-SY5Y cells exposed to TauO alone showed significant cell shrinkage and cell death as we observed cells positive to Apopxin signal and few also to 7-AAD and an overall decreased number of live cells (blue) compared to either the control cells (Ctrl) or those cells that were treated with TauO in the presence of curcumin-derived small molecules. Altogether, these observations suggest that the selected curcumin derivatives interact and subsequently convert the toxic TauO into higher molecular weight aggregates, thereby modulating their toxicity.

**Figure 7:**
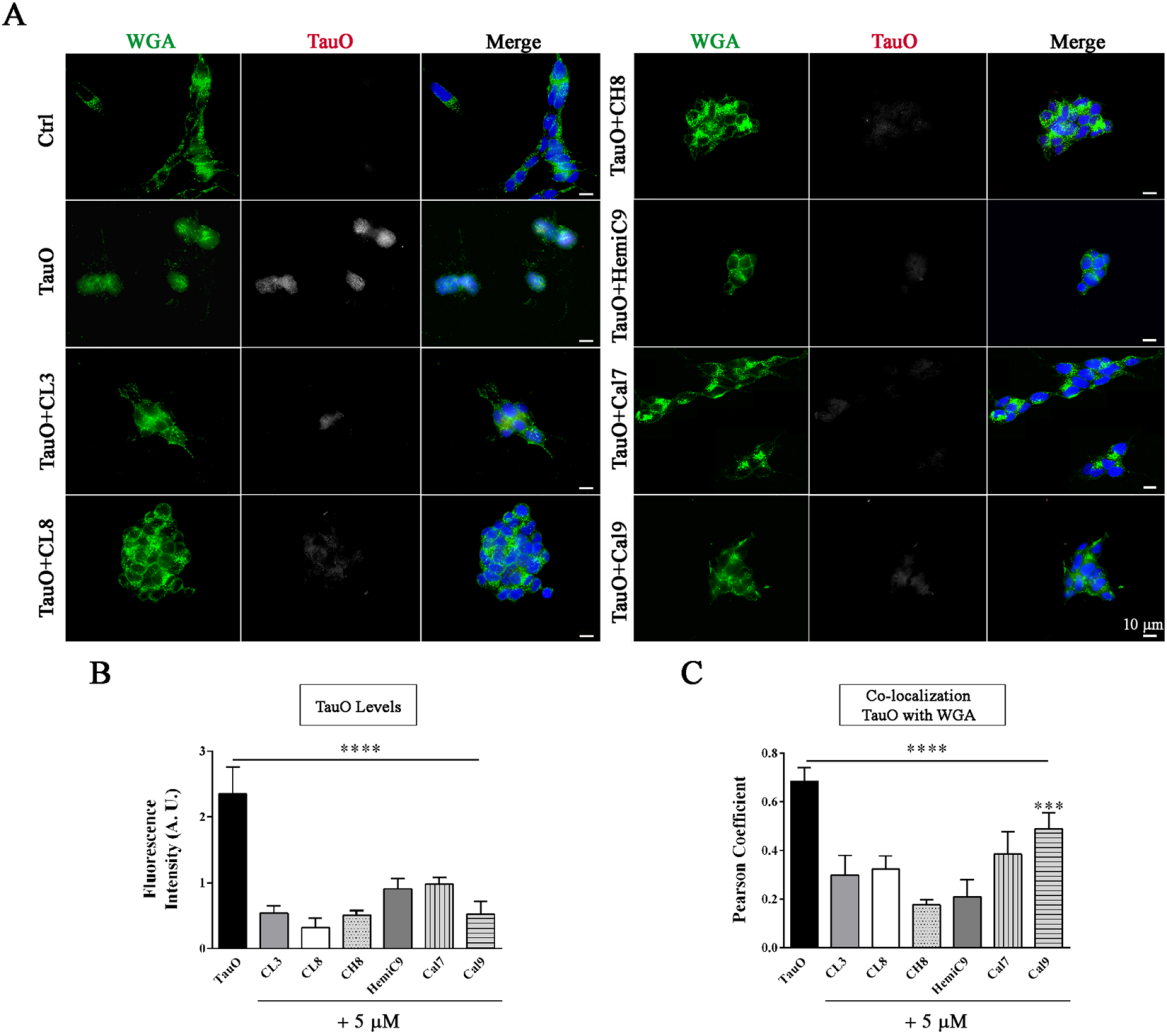
Representative epifluorescence images of human SH-SY5Y neuroblastoma cells after treatment with TauO or TauO in the presence curcumin derivatives. (**A**) SH-SY5Y cells were treated for 2 hours with sub-lethal concentration (0.5 µM) of TauO in the absence and presence of curcumin derivatives (final concentration 5 µM). Cells were immunostained with DAPI (nuclei – blue), Wheat Germ Agglutinin (WGA) – Alexa Fluor-488 (plasma membranes – green) and TauO labeled with Alexa Fluor-568 (red represented in grey). Scale bar 10 μm. (**B**) Curcumin derivative-induced aggregates are taken up less by the cells, as shown by the decreased fluorescence intensity of TauO in the presence of curcumin derivatives as compared to TauO. (**C**) PCC graph represents co-localization coefficient of TauO with WGA, showing a significant reduction of TauO in the plasma membranes when cells are exposed to TauO in the presence of compounds. Analyses were performed on selected regions of interest characterized by same size and comparable number of cells. Data in B and C were compared by one-way analysis of variance (ANOVA) followed by Dunnett’s multiple comparison test: ***p < 0.001, ****p < 0.0001. Bars and error bars represent means and standard deviations performed.

**Figure 8:**
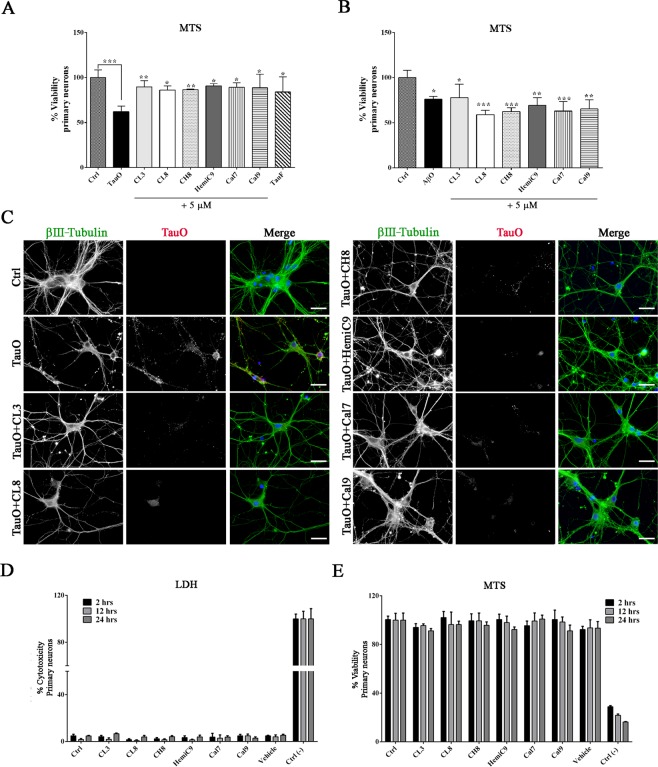
Curcumin derivatives prevent TauO-induced toxicity in primary cortical neurons. (**A**)Viability percentage of neuronal culture exposed to 0.5 µM of TauO or 0.5 µM of TauO pre-incubated with 5 µM curcumin derivatives and controls for 2 hours assessed by MTS assay. Neurons exposed to TauO pretreated with curcumin derivatives had significantly higher cell viability as compared to those exposed to untreated TauO. **(B)** Primary neurons exposed to 0.5 µM of Aβ oligomers, 0.5 µM of Aβ oligomers pre-incubated with 5 µM curcumin derivatives and controls. Neuronal cells treated with AβO in the presence of curcumin derivatives show no changes in cell viability as compared to untreated AβO, suggesting that the selected curcumin derivatives are not able to rescue cells from AβO-induced toxicity. (**C**) Representative epifluorescence images of cortical neurons exposed to TauO labeled with Alexa Fluor-568 or TauO labeled with Alexa Fluor-568 in the presence of curcumin analogs. Neurons were immunostained with βIII–Tubulin (mature neurons – green represented in grey), TauO (red represented in grey) and DAPI (nuclei – blue). Scale bar = 20 µm. (**D-E**) Cytotoxicity and viability percentage of neuronal culture exposed to 5 µM of curcumin derivatives over time (2, 12 and 24 hours). Data in A, B, D and E were compared by one-way analysis of variance (ANOVA) followed by Dunnett’s multiple comparison test: *p < 0.05; **p < 0.01; ***p < 0.001. Bars and error bars represent the mean and standard deviation (n = 3).

In addition, to further confirm our findings and gain a better understanding of the protective role of curcumin derivatives, internalization screens were carried out in human neuroblastoma cell culture. SH-SY5Y cells were treated with a sub-lethal concentration (0.5 μM) of tau oligomers in the absence and presence of curcumin compounds and then imaged by fluorescence microscopy (Fig. 7). Tau oligomers were observed in both the plasma membranes and in the cytoplasm, indicating extensive cellular internalization of TauO. Furthermore, cells exposed to untreated TauO, exhibit extensive loss of plasma membrane integrity, reflecting the toxic effect of tau oligomers. Excitingly, cells that were treated with TauO in the presence of curcumin derivatives show a significant reduction in the percentage of area positive of TauO staining (Fig. 7B). Immunofluorescence analysis showed that the tau species, resulting from the incubation with the curcumin derivatives, mostly co-localize with the plasma membrane, as shown by PCC graph (Fig. 7C). Altogether, the data implies that curcumin derivatives-induced aggregates are less prone to be internalized by the cells, thereby elucidating their reduced cytotoxicity. The toxicity of the curcumin derivatives-induced aggregates was also evaluated by using primary cortical neurons isolated from embryos of Htau mice, expressing non-mutant human tau. Primary neurons are an approximate representation of the cellular physiology, genetic expression and protein profile *in vivo*, providing a more appropriate model for drug targeting validation. Therefore, neurons were exposed to tau oligomers alone or in the presence of curcumin derivatives (Fig. 8). Cell viability significantly decreased after treatment with untreated TauO, while treatment with curcumin derivatives significantly reduced their toxicity as seen by the higher level of cell viability, as assessed by MTS assay (Fig. 8A). Furthermore, curcumin derivatives were also incubated with Aβ oligomers (AβO). The toxicity screens in primary neurons showed that the curcumin compounds were not able to rescue neurons from Aβ oligomer-induced toxicity (Fig. 8B). Moreover, to further investigate and gain an enhanced understanding of curcumin derivatives effects, primary cortical neurons were treated with TauO labeled with Alexa Fluor-568, or TauO labeled with Alexa Fluor-568 in the presence of curcumin derivatives. Cortical neurons were immunostained with the mature neuronal marker β-III Tubulin (green) and DAPI for nuclei (blue) and imaged by fluorescence microscopy (Fig. 8C). Interestingly, TauO were observed in the cell bodies and projections of neurons exposed to untreated TauO, indicating that TauO were extensively internalized by the neurons. On the other hand, immunofluorescence images of neurons treated with TauO in the presence of curcumin derivatives show a significant reduction in area positive to TauO, suggesting that the resulting tau aggregates are less readily internalized by the cells, thus confirming their reduced associated neurotoxicity. In addition the effects of the selected curcumin analogs have been evaluated in primary cortical neurons at 2, 12 and 24 hours showing to have no toxic effects at 5 µM as assessed by LDH and MTS assay (Fig. 8D,E).

## Discussion

The large body of evidence supporting the key role of tau in neurodegenerative diseases suggests the importance of tau as a potential target for the development of successful disease-modifying therapeutics. Unfortunately, the aggregation of proteins and their tendency to propagate makes the treatment of tauopathy difficult. As a result, there is urgent need to diagnose these disorders earlier, and to be proactive with treatment prior to any significant spreading of the neurodegenerative pathology. To date, most drug development for tauopathies are focused primarily on tau aggregation inhibitors or small molecules with disassembling potency^34,62,63^. Investigations regarding treatment options should also be directed toward small molecules capable of targeting the toxic tau species and promoting the formation of a non-toxic aggregation state, or perhaps conformations that can be more readily degraded by active cellular mechanisms such as autophagy and proteasomal degradation^64–68^.

With the strong evidences supporting that the soluble, oligomeric tau are likely the most toxic species causing disease, the development of effective interventions and therapeutics to stave or reverse their propagation has become paramount^69–73^. Accelerating the fibrillization process has the potential for being an alternative approach, considering that it was previously used in the Aβ field^74^. However, it is important to confirm that these approaches do not inhibit fibril formation at the cost of stabilizing the toxic oligomer, as seen in many cases^75^. Therefore, in this study, we investigated the effects of newly synthesized curcumin derivatives on toxic tau oligomers. Herein, using fully synthetic curcumin derivatives, we report the new finding that curcumin-derived small molecules can interact with the highly toxic tau species. Our results suggest that the selected six curcumin analogs modulate the aggregation state of toxic tau oligomers by further promoting their aggregation, resulting in the formation of larger tau structures with decreased toxicity. Indeed, toxicity and internalization screens were assessed using both human neuroblastoma SH-SY5Y cell lines and cultured primary cortical neurons. Treatment with the selected active curcumin derivatives protected SH-SY5Y and primary cortical neurons from tau oligomer-induced neurotoxicity, while the same compounds were not able to prevent neurons from AβO-induced toxicity. In addition, internalization screens showed that the compounds affect the tau oligomer internalization mechanism that mediates their uptake by cells, a critical step for the cell-to-cell spreading and the further progression of the pathology. Importantly, the present study suggests that modulating the aggregation state of toxic tau oligomeric species through the use of small molecules, including our promising active curcumin derivatives, could be a powerful therapeutic strategy that targets their toxicity regardless of the multitude of factors that may be involved in the formation of tau oligomers.

In conclusion, the results presented here lay the groundwork for future studies to test the efficacy and beneficial effects of some curcumin derivatives towards treating tauopathies. By expanding our investigation to disease-relevant tau oligomers,  derived from brain tissues of different tauopathies, and the effects of curcumin derivatives in animal models of tauopathies, will further increase our understanding of the therapeutic potential and the mechanism of action of curcumin derivatives. Our results also suggest that curcumin derivatives may be used as potential tau PET tracers for the early detection of tau oligomers. Indeed, they can be used to stabilize the highly dynamic and transient oligomers into larger and stable tau aggregates such that they may be employed for diagnostic purposes as imaging agents to enhance the weak imaging signals of small oligomers, providing opportunity for prompt interventions.

## Methods

### Chemistry

All solvent and reagents were used as received, unless otherwise stated. Melting points were determined on a hot-stage apparatus. ^1^H-NMR and ^13^C-NMR spectra were recorded at indicated frequencies, residual solvent peak was used as reference. Chromatography was performed by using silica gel (0.040–0.063 mm) and mixtures of ethyl acetate and petroleum ether (fraction boiling in the range of 40–60 °C) in various ratios (v/v). All solvent and reagents were used as received. Compounds **CL3**^58^, and **HemiC9**^76^, were prepared as previously reported. **CL8**^58^**, CH8**^54,60^, and **Cal7,9**^61^ were prepared adapting previously reported methods. Synthetic details and spectroscopic characterization for all compounds are reported on SI.

### Biology

Animal handling and all the experimental procedures were performed in accordance with the Guide for the Care and Use of Laboratory Animals (National Institutes of Health) and accordingly to the relevant guidelines and regulations approved by the Institutional Animal Care and Use Committee of the University of Texas Medical Branch (UTMB). Mice were housed at the UTMB animal care facility and maintained according to the U.S. Department of Agriculture standards (12 hours light/dark cycle with ad libitum access to water and food).

### Preparation of tau oligomers

Recombinant tau protein (tau-441 (2N4R) MW 45.9 kDa) was expressed and purified as described^77,78^. The tau pellet was treated with 8 M urea followed by overnight dialysis against 1X phosphate-buffered saline (PBS) pH 7.4. Tau concentration was measured using bicinchoninic acid protein assay (Pierce BCA Protein Assay Kit, 23225, Thermo Fisher Scientific) and normalized to 1 mg/mL by adding 1X PBS. Aliquots of tau monomer in PBS were stored at -20 °C. Each 300 µL of tau stock (0.3 mg) was added to 700 µL of 1X PBS and incubated for 1 hour on an orbital shaker at room temperature. After shaking, the resulting tau oligomers were purified by fast protein liquid chromatography (FPLC, Superdex 200 Increase 10/300 column, Amersham Biosciences).

### Preparation of tau fibrils

Recombinant tau protein (tau-441 (2N4R) MW 45.9 kDa) was expressed and purified as described^77,78^. The tau pellet was treated with 8 M urea followed by overnight dialysis against 1X phosphate-buffered saline (PBS) pH 7.4. Tau concentration was measured using bicinchoninic acid protein assay (Pierce BCA Protein Assay Kit, 23225, Thermo Fisher Scientific) and normalized to 1 mg/mL by adding 1X PBS. Aliquots of tau monomer were incubated with heparin (15 kDa) (1:5 molar ratio) at 37 °C on an orbital shaker at a speed of 30 rpm for 5 days as previously described^79^.

### Preparation of tau oligomers in the presence of curcumin and curcumin derivatives

A volume of 100 μL of tau oligomers (1 µg/μL) was incubated with curcumin (1:5 and 1:10 molar ratio) and curcumin derivatives (1:5 molar ratio). Compounds were dissolved in EtOH 75%/DMSO (5:1) at a final concentration of 5 mM and diluted in 1X PBS or ddH_2_O for incubation or toxicity assay (final concentration 5 μM). Tau oligomers in the presence of the small molecules and controls were incubated on an orbital shaker, without stirring, for 16 hours under oligomerization conditions as previously described^64^.

### Preparation of Aβ oligomers

Aβ oligomers (AβO) were prepared as previously described^80^. An amount of 0.3 mg of Aβ pellet was dissolved in 200 μL of hexafluoroisopropanol (HFIP) and incubated for 10–20 min at room temperature. The resulting solution was added to 700 μL of ddH_2_O in a siliconized Eppendorf tube with holes placed on top of the cap to allow the slow evaporation of HFIP. The samples were then stirred at 500 rpm using a Teflon-coated micro stir bar for 48 hours at room temperature in the fume hood.

### Preparation of Aβ oligomers in the presence of curcumin derivatives

A volume of 100 μL of Aβ oligomers (0.5 µg/μL) was incubated with curcumin derivatives (final concentation 5 μM). Compounds were dissolved in EtOH 75%/DMSO (5:1) at a final concentration of 5 mM and diluted in 1X PBS or ddH_2_O for incubation or toxicity assay (final concentration 5 μM). Aβ oligomers in the presence of the small molecules and controls were incubated on an orbital shaker, without stirring, for 16 hours under oligomerization conditions as previously described^64^.

### Western blotting

An amount of 3 µg of each sample were resolved on a pre-cast NuPAGE 4–12% Bis-Tris Gels for SDS-PAGE (NP0335BOX, Invitrogen) and transferred to nitrocellulose membranes. Then, membranes were blocked with 10% nonfat milk in Tris-buffered saline with very low tween 0.01% (TBS-T) overnight at 4 °C. After blocking, membranes were probed with T22 (1:250) for tau oligomers, Tau 5 (1:10000; 806402, BioLegend) and Tau 13 (1:50.000; MMS-520R, Biolegend) for total tau, and horseradish peroxidase (HRP) anti-β-Amyloid, 6E10 for total Aβ (1:1000; 9345–02, Covance) diluted in 5% nonfat milk for 1 hour at RT. Membranes were then incubated with HRP-conjugated IgG anti-rabbit (1:10000, GE Healthcare) to detect T22 and anti-mouse (1:10000, GE Healthcare) secondary antibody to detect Tau 5 and Tau 13. ECL plus (GE Healthcare) was used for signal detection.

### Dot Blot

Dot blot assay to detect tau oligomers in the absence or presence of small molecules was performed as previously described^80^. Briefly, 1.5 μl of each end-product reaction was applied onto nitrocellulose membranes and then blocked with 10% nonfat milk in TBS-T overnight at 4 °C. The next day, membranes were probed with the oligomer-specific tau antibodies, T22 (1:250), TOMA1(1:200) and total tau antibody, Tau 5, (1:10000) diluted in 5% nonfat milk for 1 hour at RT. Membranes were then incubated with HRP-conjugated IgG anti-rabbit (1:10000) to detect T22 and anti-mouse (1:10000) secondary antibody to detect Tau 5 and TOMA1. Blots were then washed three times in TBS-T and ECL plus (GE Healthcare) was used for signal detection.

### Filter Trap Assay

Filter Trap assay was performed using Bio-Dot SF Microfiltration Apparatus (Bio-Rad), as previously described^64^. Briefly, 1 μg of each end-product reaction was applied onto nitrocellulose membranes, previously pre-wetted with TBS-T, through the use of a vacuum based bio-slot apparatus. Membranes were then blocked with 10% nonfat milk in TBS-T overnight at 4 °C. Next day, membranes were probed with the oligomer-specific tau antibodies, T22 (1:250) and TOMA1(1:200) and total tau antibody, Tau 5 (1:10000) diluted in 5% nonfat milk for 1 hour at RT. Membranes were then incubated with HRP-conjugated IgG anti-rabbit (1:10000) to detect T22 and anti-mouse (1:10000) secondary antibody to detect Tau 5 and TOMA1. Membranes were then washed three time in TBS-T and ECL plus (GE Healthcare) was used for signal detection.

### Direct ELISA

ELISA assay was conducted as previously described^80^. Briefly, 96 well plates (Nunc Immobilizer, Amino Plates and Modules, 436006, Thermo Fisher Scientific) were previously coated with 1.5 μL of tau oligomers in the presence or absence of curcumin derivatives using 50 μL of 1X PBS, pH 7.4, as coating buffer. After washing three times with TBS-T, plates were blocked for 2 hours at room temperature with 120 µL of 10% nonfat milk in TBS-T. Plates were then washed three times with TBS-T, and probed with 100 μL of primary antibodies for 1 hour at room temperature, T22 (diluted 1:250 in 5% nonfat milk in TBS-T) and Tau 5 (diluted 1:10000 in 5% nonfat milk in TBS-T). Plates were then washed three times with TBS-T and incubated with 100 μL of HRP-conjugated anti-rabbit or anti-mouse IgG, diluted 1:10000 in 5% nonfat milk in TBS-T, for 1 hour at room temperature. Plates were washed three times with TBS-T and developed with 3,3,5,5-tetramethylbenzidine (TMB + Substrate- Chromogen, S1599, Dako) The reaction was stopped using 100 μL of 1 M HCl and absorbance was read at 450 nm using POLARstar OMEGA plate reader. All experiments were performed in triplicate.

### Sandwich ELISA

Sandwich ELISA assay was conducted as previously described^81^. Briefly ELISA plates (Nunc Immobilizer Amino Plate, 442404, Thermo Fisher Scientific) were coated with the capture antibody, T22 (1:250) diluted in sodium bicarbonate buffer, pH 9.6. The plate was incubated at 4 °C overnight. The following day, after washing two times with TBS-T, plates were blocked for 2 hours at 37 °C with 120 µL of 10% nonfat milk in TBS-T. The plate was then loaded with 2 µg of recombinant tau oligomers in PBS and added to each well for 90 minutes at 37 °C. Plates were then washed three times with TBS-T, and probed with 100 μL of anti-tau antibody, Tau 5 (diluted 1:10000 in 5% nonfat milk in TBS-T) for 1 hour at room temperature. After washing three times with TBS-T, plate were incubated with of 100 μL of HRP-conjugated anti-mouse IgG, diluted 1:10000 in 5% nonfat milk in TBS-T, for 1 hour at room temperature. Plates were washed three times with TBS-T and developed with 3,3,5,5-tetramethylbenzidine (TMB + Substrate- Chromogen, S1599, Dako). The reaction was stopped using 100 μL of 1 M HCl and absorbance was read at 450 nm using POLARstar OMEGA plate reader. All experiments were performed in triplicate.

### Size exclusion chromatography

Tau oligomers alone and in the presence of curcumin derivatives were analyzed using AKTA Explorer system fitted with a Superdex 200 Increase 10/300 GL Column. Degassed deionized water was used as mobile phase with a flowrate of 0.5 mL/min. Gel filtration standard (Bio-Rad 51-1901) was used for calibrations. Samples were resolved using absorbance at 280 nm.

### Morphological analysis of TauO by AFM

Tau oligomers were characterized by AFM as previously described^79,80,82^. Briefly, samples were prepared by adding 5 µL tau oligomers in the absence or presence of curcumin derivatives onto freshly-cleaved mica and allowed to adsorb to the surface. Mica were then washed three times with deionized water to remove unbound protein and impurities and then air-dried. Samples were then imaged with a Multimode 8 AFM machine (Veeco, CA) using a non-contact tapping method (ScanAsyst-Air). AFM analyses were performed using the particles analysis tool of the NanoScope Analysis v1.20rl AFM data processing software.

### Bis-ANS and Thioflavin T (ThT) Fluorescence

Fluorescence spectroscopy was performed as previously described^64^. Samples were prepared by adding 2 µL of protein (0.3–0.5 µg/µL) and 248 µL of 10 µM bis-ANS (4,4′-dianilino-1,1′-binaphthyl-5,5′-disulfonic acid)(B153, Invitrogen), prepared in 100 mM glycine-NaOH buffer (pH 7.4), in a clear bottom 96-well black plate. Each experiment was performed in triplicate. The bis-ANS fluorescence intensity was measured at an emission wavelength of 520 nm upon excitation at 380 nm. For the ThT assay, samples were prepared using 2 µL of protein (0.3–0.5 µg/µL) and 248 µL of 5 µM ThT (T3516, Sigma), dissolved in 50 mM glycine-NaOH buffer (pH 8.5). Each experiment was performed in triplicate. ThT fluorescence intensity was recorded at an emission wavelength of 490 nm upon excitation at 440 nm using a POLARstar OMEGA plate reader (BMG Labtechnologies). Fluorescence spectra of the following solutions were measured as negative controls for both dyes (bis-ANS and ThT): dye alone, dye + vehicle. In addition, fluorescence spectra of dye + curcumin derivatives were measured to avoid any false positive readings due to the eventually intrinsic fluorescent properties of curcumin derivatives. Each reading was corrected for the corresponding background fluorescence.

### Primary cortical neurons

Primary cortical neurons from transgenic mice expressing human full-length tau were prepared and maintained as previously described^83^. Briefly, cortical neurons were isolated from embryos at embryonic day 16–18 using Accutase solution (A6964, Sigma). Dissociated neurons were plated at a density of 30 × 10^4^ cells/well in 96-well plates containing high glucose Dulbecco’s Modified Eagle Medium (DMEM;10–013-CV, Corning) supplemented with 2% B27 (A3582801, Gibco), 10,000 units/mL penicillin, 10,000 μg/mL streptomycin, and 25 μg/mL Amphotericin B (Gibco). After 2 hours, plating medium was removed from cells and replaced with Neurobasal medium (12348017, Gibco) plus 2% B27, 0.5 mM GlutaMax (35050-061, Gibco), 10,000 units/mL, 10,000 μg/mL streptomycin, and 25 μg/mL Amphotericin B supplement. Cells were grown for 10–12 days *in vitro* before experiments and 50% of media changes were performed every 3 days. On day 10, neuronal cultures were treated with 0.5 μM tau oligomers alone and in the presence of curcumin derivative (at final concentration 5 μM) for two hours. Cell viability was assessed by MTS assay.

### Cell toxicity assays

#### MTS

Human neuroblastoma SH-SY5Y cells (ATCC CRL-2266) were maintained in DMEM and grown to confluence in 96-well plates as previously described^64^. Cells (≈10,000 cells /well) were treated with 2.0 µM tau oligomers and 2.0 µM tau oligomers incubated with 5 µM of curcumin or curcumin derivatives for 24 hours. The dose-response curve was used to calculate the concentration of the compound required for 50% inhibition of cell viability (IC_50_).

Primary neurons were treated with 0.5 µM tau oligomers or 0.5 µM tau oligomers pre-incubated with 5 µM of curcumin derivatives for 2 hours. Primary neurons were also treated with 5 µM of curcumin derivatives and cell viability was assessed over the time at 2, 12 and 24 hours by evaluating LDH release of the cells.

Cell viability was corrected by the vehicle background. All measurements were performed in triplicate. The cytotoxic effect was determined using MTS assay (G3582, Promega) for assessing cell viability following the manufacturers’ instructions. Optical density (OD) was measured at 490 nm with POLARstar OMEGA plate reader (BMG Labtechnologies). Cell viability was calculated as the percentage of the OD value of treated cells compared to untreated controls, according to the following equation: Viability = (OD SAMPLE/OD CONTROL) × 100%.

### LDH

Human neuroblastoma SH-SY5Y cells were cultured and treated for measuring cytotoxicity using LDH release assay (Cytotoxicity Detection KitPLUS-LDH, 04744926001, Roche) following the manufacturers’ instructions as previously described^80,84,85^. Briefly, cells were maintained in DMEM and grown to confluence in 96-well plates. Cells (≈10,000 cells /well) were treated for 24 hours with 2.0 µM TauO or 2.0 µM TauO incubated with curcumin derivatives (final concentration 5 µM) and assessed by LDH assay.

Primary neurons were treated with 5 µM of curcumin derivatives and assessed their cytotoxicity over the time at 2, 12 and 24 hours by evaluating LDH release.

OD was measured at 490 nm with POLARstar OMEGA plate reader (BMG Labtechnologies). All measurements were performed in triplicate and corrected by the vehicle background.

### Immunofluorescence – SH-SY5Y

SH-SY5Y cells were maintained in DMEM and grown to confluence using poly-L-lysine coated coverslip in 24-well plates as previously described^86,87^. Cells (≈20,000 cells /well) were treated for 1 hour with 0.5 µM TauO labeled with Alexa Fluor-568 or 0.5 µM TauO labeled with Alexa Fluor-568 (A20003, Invitrogen) pretreated with 5 µM of curcumin derivatives. After washing off unbound proteins, cells were immunostained with 5 μg/mL Wheat Germ Agglutinin (WGA) Alexa Fluor-488 (W11261, Invitrogen) for 10 min followed by fixation in chilled methanol. After washing three times with 1X PBS, cells were permeabilized with 0.25% Triton-X 100, diluted in 1X PBS for 10 min. Cells were then washed three times with 1X PBS (10 min each) and mounted using Prolong Gold Antifade mounting media with DAPI (P36935, Invitrogen). Slides were then dried in fume hood. Cells were imaged with Keyence BZ-800 Microscope using standard filters for DAPI, GFP and Texas Red channels and analyses have been conducted using BZ-X Analyzer software. Nikon 100X oil immersion objective was used to capture images.

### Apoptosis analysis – fluorescence microscopy assay

SH-SY5Y cells were maintained in DMEM and grown to confluence using poly-L-lysine coated coverslip in 24-well plates as previously described^86,87^. Cells (≈20,000 cells /well) were treated for 24 hours with 2 µM TauO or 2.0 µM TauO incubated with curcumin derivatives (final concentration 5 µM). After washing off unbound proteins, cellular apoptosis/necrosis was evaluated by using an Apoptosis/Necrosis Detection kit (ab176749, Abcam). Cells were incubated with Apopxin to detect apoptosis, 7-AAD for necrosis and CytoCalcein Violet 450 to detect live cells, for 30 min at room temperature. After washing, cells were imaged with Keyence BZ-800 Microscope using standard filters for DAPI, GFP and Texas Red channels. Nikon 20X objective was used for capturing images.

### Immunofluorescence – primary cortical neurons

Primary cortical neurons from C57BL/6 mice (Jackson Laboratory, stock#000664) were plated at a density of 1 × 10^6^ cells/mL using poly-L-lysine coated coverslip in 24-well plates as previously described^83^. Cortical neurons were treated for 1 hour with 0.5 µM TauO labeled with Alexa Fluor-568 or 0.5 µM TauO labeled with Alexa Fluor-568 pretreated with 5 µM of curcumin derivatives. After washing off unbound proteins, cells were fixed in 4% paraformaldehyde for 15 min at room temperature. Neurons were then washed three times with 1X PBS (10 min each) and permeabilized with 0.25% Triton-X 100, diluted in 1X PBS for 10 min. After washing three times with 1X PBS, neurons were blocked with 5% goat serum for 1 hour. Neurons were then immunostained overnight with the mature neuronal marker, β-III Tubulin (1:1000; ab78078, Abcam) at 4 °C. The next day, neurons were washed three times with1X PBS and incubated with goat anti-mouse IgG Alexa Fluor-488 (1:500, Thermo Fisher Scientific) for 1 hour at room temperature. After washing with 1X PBS, neurons were mounted using Prolong Diamond Antifade mounting media with DAPI (P36966, Invitrogen). Slides were then dried in fume hood. Cortical neurons were imaged with a Keyence BZ-800 Microscope using standard filters for DAPI, GFP and Texas Red. Nikon 100X oil immersion objective was used to capture images.

### Statistical analysis

Western blot and filter trap assay signals were quantified using ImageJ. All data collected from Western blot, filter trap assay, ELISA as well as cell toxicity assays, were subjected to statistical analyses and compared by one-way analysis of variance (ANOVA) followed by Dunnett’s multiple comparison test using GraphPad Prism 6.01 software. Results were considered statistically significant at p < 0.05. Each experiment was performed in triplicate (n = 3). Image J was used for immunofluorescence quantifications and integrated densities were plotted as bar graphs expressed as mean ± standard deviation. To measure co-localization from immunofluorescence, we used Pearson Correlation Coefficient (PCC). Analyses were conducted for each condition in five different selected regions of interest characterized by same size and comparable number of cells. All data collected from fluorescence assays (Immunofluorescence, bis-ANS and Thioflavin T spectroscopy binding assays) were subjected to statistical analyses and compared by one-way analysis of variance (ANOVA) followed by Dunnett’s multiple comparison test using GraphPad Prism 6.01 software.

## Supplementary information

Supplementary Information

## Data Availability

The data that support the findings of this study are available from the corresponding author upon reasonable request.
