# Supplementary material for: Toxic Tau Oligomers Modulated by Novel Curcumin Derivatives
Source: Sci Rep. 2019 Dec 12;9:19011. doi: 10.1038/s41598-019-55419-w (PMC6908736; doi:10.1038/s41598-019-55419-w)
Supplement: Supplementary file 1 — Supplementary Information [file 41598_2019_55419_MOESM1_ESM.pdf]

Supplementary information

Toxic Tau Oligomers Modulated by Novel Curcumin Derivatives

Filippa Lo Cascio[1,2], Nicha Puangmalai[1,2], Anna Ellsworth[1,2], Fabio Bucchieri[3], Andrea Pace[4], Antonio Palumbo Piccionello[4], Rakez Kayed[1,2,†]

[1]Mitchell Center for Neurodegenerative Diseases, University of Texas Medical Branch, Galveston, TX, 77555, USA

[2]Departments of Neurology, Neuroscience and Cell Biology, University of Texas Medical Branch, Galveston, TX, 77555, USA

[3]Department of Experimental Biomedicine and Clinical Neuroscience, University of Palermo, Italy

[4]Department of Biological, Chemical and Pharmaceutical Sciences and Technologies-STEBICEF, University of Palermo, Italy.

† To whom correspondence should be addressed: *rakayed@utmb.edu*

Table of Contents

Figure S1 .... S2
Figure S2 .... S3
Figure S3 .... S4
Figure S4 .... S5
Experimental Section S6
Hemi-curcuminoid derivatives 2-5 .... S6
Scheme S1 .... S6
Table S1 .... S7
Cinnamils (1,6-diarylhexa-1,5-diene-3,4-diones) CL S7
Scheme S2 .... S7
1,2,4-oxadiazole derivatives CH1-4 .... S8
Scheme S3 .... S8
1,3,4-oxadiazole derivatives CH5-11 .... S8
Scheme S4 .... S8
Calebin-A analogs Cal1-9 .... S9
Scheme S5 .... S9
General methods S10
References S17

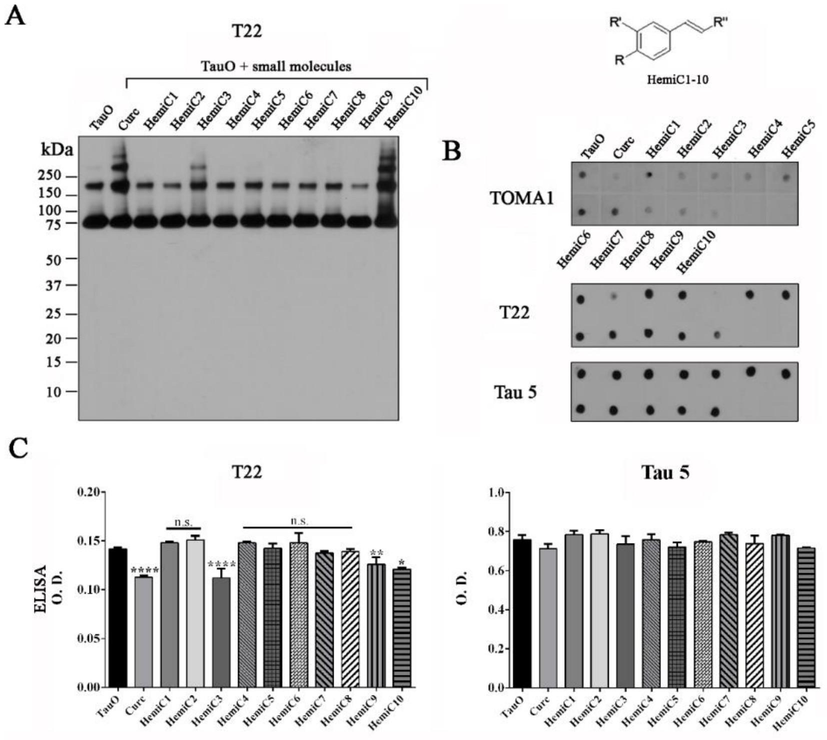

Figure S1. Biochemical analysis of oligomeric tau treated with Hemi-curcuminoid (**HemiC**) derivatives and untreated control. (**A**) Western blot analysis of tau oligomers alone and pretreated with curcumin and Hemi-curcuminoid analogs probed with T22, shows that some of the compounds can alter the aggregation states of preformed tau oligomers. (**B**) Dot Blots analysis of oligomeric tau alone and in the presence of **HemiC**, probed with the Tau Oligomer Monoclonal Antibody (TOMA1), T22 and Tau5, shows that some of the **HemiC** compounds are able to decrease tau oligomer levels as compared to the untreated control. (**C**) ELISA analysis of oligomeric tau with and without **HemiC** analogs shows that some **HemiC** affect tau aggregation pathways reducing tau oligomer levels as compared to the untreated control while there is no change in total tau protein as assessed by Tau 5 immunoreactivity. Data were compared by one-way analysis of variance (ANOVA), followed by Dunnett's multiple comparison test: *p<0.05; **p<0.01; ****p<0.0001. Bars and error bars represent the mean and standard deviation.

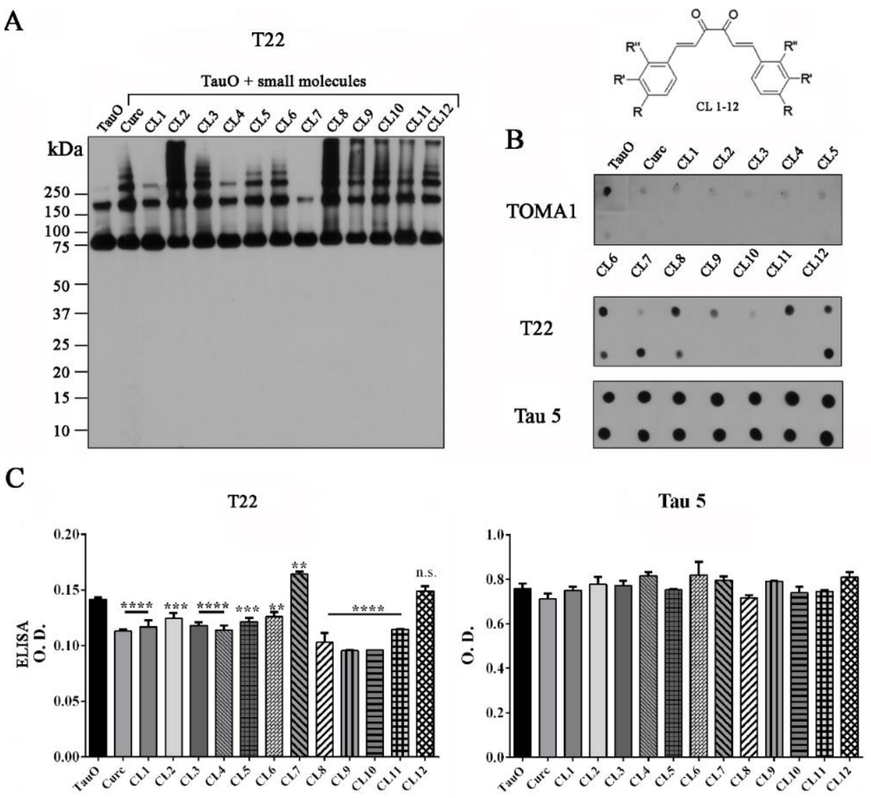

Figure S2. Biochemical analysis of oligomeric tau treated with Curcumin-like (**CL**) derivatives and untreated control. **(A)** Western blot analysis of tau oligomers alone and those pretreated with curcumin and **CL** analogs, probed with T22, shows that the compounds are able to alter the aggregation states of preformed tau oligomers. **(B)** Dot blot analysis probed with anti-oligomeric monoclonal and polyclonal tau antibodies, respectively TOMA1 and T22, and total tau antibody, Tau 5. **(C)** ELISA analysis of oligomeric tau shows a significant decrease in the tau oligomer levels in the presence of the **CL** compounds as compared to the untreated control, TauO. Data were compared by one-way analysis of variance (ANOVA), followed by Dunnett's multiple comparison test: $^{**}p<0.01$; $^{***}p<0.001$; $^{****}p<0.0001$. Bars and error bars represent the mean and standard deviation.

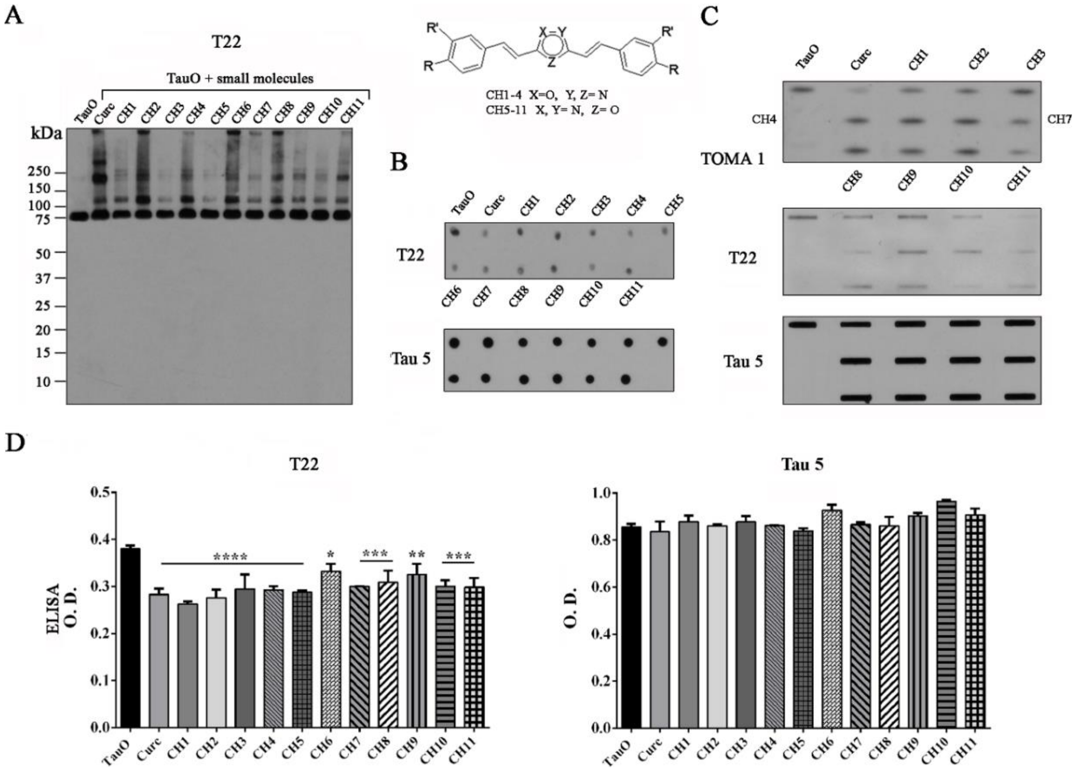

Figure S3. Biochemical analysis of oligomeric tau with and without Heterocyclic curcumin (**CH**) derivatives treatment. **(A)** Western blot analysis of tau oligomers alone and pretreated with curcumin and Heterocyclic curcumin analogs probed with T22, shows that the incubation with the compounds modulates the aggregation states of preformed tau oligomers as compared to the untreated TauO. **(B)** Filter Trap and Dot blot analyses of tau oligomers alone and pretreated with curcumin and **CH** analogs probed with T22 and Tau 5. Some of the compounds are able to alter the aggregation states of preformed tau oligomers, resulting in decreased tau oligomer levels as compared to tau oligomers alone. **CH** analogs reduce TOMA1 immunoreactivity. **(C)** ELISA analysis of oligomeric tau with and without **CH** derivatives show decreased T22 immunoreactivity after treatment with the compounds and no changes in total tau protein as assessed using Tau 5 antibody. Data were compared by one-way analysis of variance (ANOVA) followed by Dunnett's multiple comparison test: *p<0.05; **p<0.01; ***p<0.001; ****p<0.0001. Bars and error bars represent the mean and standard deviation.

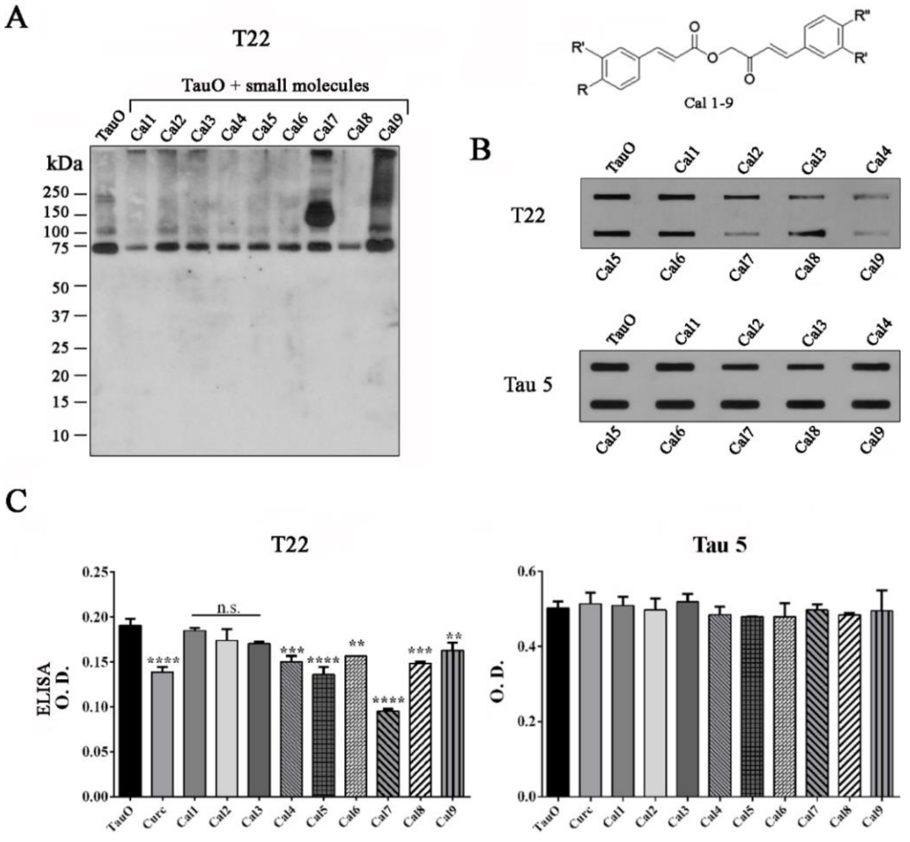

Figure S4. Biochemical analysis of oligomeric tau with and without Calebin-A (**Cal**) derivatives treatment. (**A**) Western blot analysis of tau oligomers alone and those pretreated with curcumin and Calebin-A analogs probed with T22, shows that the incubation with the compounds modulates the aggregation states of tau oligomers as compared to the untreated TauO. (**B**) Filter Trap assay, probed with T22 and Tau 5, show that some of the compounds decrease T22 immunoreactivity as compared to the untreated TauO. (**C**) ELISA analysis of oligomeric tau after treatment with **Cal** derivatives shows that some of the compounds decrease tau oligomer levels as seen by the reduced T22 immunoreactivity and no changes in total tau protein using Tau 5. Data were compared by one-way analysis of variance (ANOVA) followed by Dunnett's multiple comparison test: **p<0.01; ***p<0.001; ****p<0.0001. Bars and error bars represent the mean and standard deviation.

Experimental Section

All solvent and reagents were used as received, unless otherwise stated. Melting points were determined on a hot-stage apparatus. ^{1}H-NMR and ^{13}C-NMR spectra were recorded at indicated frequencies, residual solvent peak was used as reference. Chromatography was performed by using silica gel (0.040–0.063 mm) and mixtures of ethyl acetate and petroleum ether (fraction boiling in the range of 40–60 °C) in various ratios (v/v). All solvent and reagents were used as received. Compounds **2a,b,e,g**[1], **2c**[2], **2d**[3], **3a,b,e,g**[1], **4b-e,j**[4], **4k**[5], **5a**[6], **5h**[7], **CL1-3,5**[8], **7**[5,9], **8**[5,10] , **CH4**[5] were prepared as previously reported. Other already known and new compounds were prepared adapting previously reported methods as indicated below.

Hemi-curcuminoid derivatives 2-5

These compounds were obtained by adapting previously reported condensation reactions (Scheme S1). *E*–α,β-Unsaturated ketones **2** were obtained through Claisen-Schmidt Aldol condensation[11] by treating commercial aldehydes **1** with acetone under basic conditions. In turn, reaction of compounds **2** with iodine, in the presence of CuO as catalyst, yields to iodo-derivatives **3**[1] *E*-Cinnamic acids **4** were obtained performing Doebner modification of Knoevenagel condensation[4], ethyl cinnamate **4k** was similarly obtained[5]. Cinnamonitriles **5a,h** were obtained from benzaldehyde **1** condensation with acetonitrile, as previously reported[7].

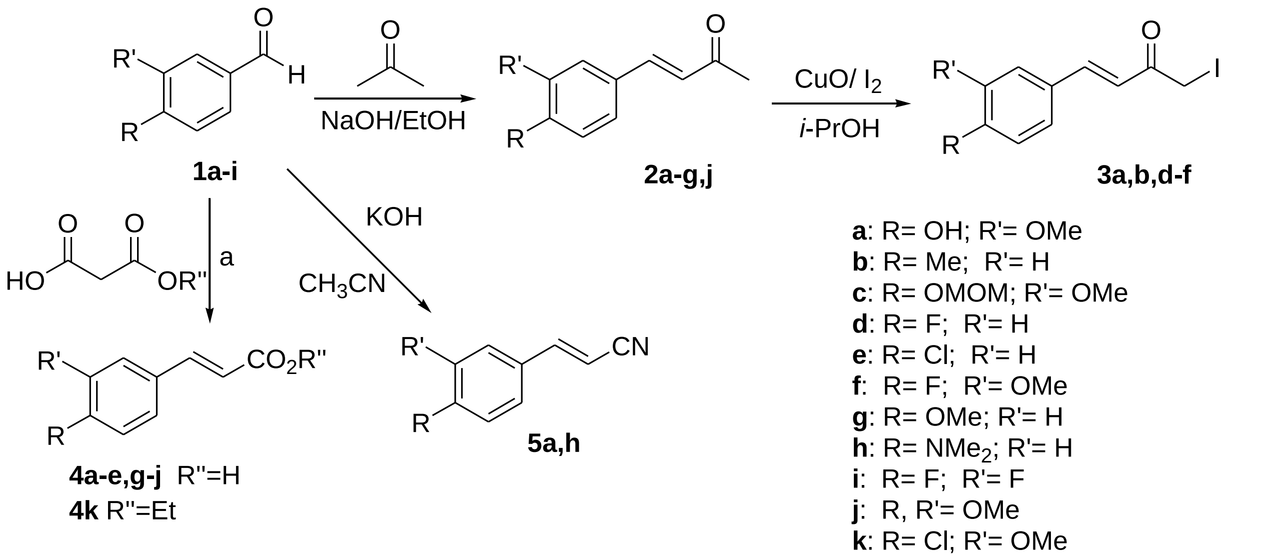

Scheme S1. Synthesis of hemi-curcuminoid compounds. (a) R″=H: pyridine, aniline (cat.), toluene, reflux; R″=Et: pyridine, piperidine, reflux.

Among obtained compounds **2-5** were selected Hemi-curcuminoid compounds **HemiC1-10** (Table S1) which were tested as representative example of variously substituted derivatives. On the other hand, compounds **2-5** were used as building-block for the obtainment of other target compounds (see below).

Table S1. Structures of tested Hemi-curcuminoid compounds (HemiC).

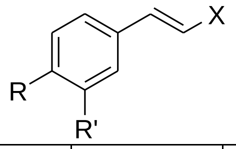

Entry ID	Compound	X	R	R′
**HemiC1**	**2a**	COMe	OH	OMe
**HemiC2**	**5a**	CN	OH	OMe
**HemiC3**	**3a**	$COCH_2I$	OH	OMe
**HemiC4**	**2g**	COMe	OMe	H
**HemiC5**	**4a**	CO_2H	OH	OMe
**HemiC6**	**2j**	COMe	OMe	OMe
**HemiC7**	**4k**	CO_2Et	OMe	Cl
**HemiC8**	**5h**	CN	NMe_2	H
**HemiC9**	**2b**	COMe	Me	H
**HemiC10**	**2c**	COMe	OMOM	OMe

Cinnamils (1,6-diarylhexa-1,5-diene-3,4-diones) CL

The synthesis of **CL1-12**, was performed through two aldol-condensation of aromatic aldehydes **1** on diacetyl **6** with the formation of both double bonds with *E* geometry (Scheme S2)[8].

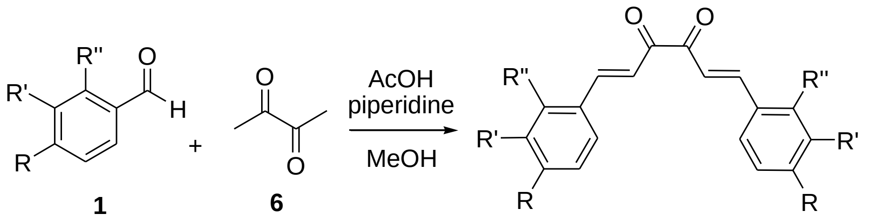

CL1: R= OMe; R',R''= H
CL2: R, R',R''= H
CL3: R= F; R',R''= H
CL4: R= NMe_2; R'= H
CL5: R= Cl; R',R''= H
CL6: R= F; R'= F
CL7: R'= Cl; R,R''= H
CL8: R''= Cl; R,R'= H
CL9: R'= OMe; R,R''= H
CL10: R= OMOM; R'= OMe; R''= H
CL11: R,R'= OCH_2O; R''= H
CL12: R= CO_2H; R',R''= H

Scheme S2. Synthesis of Cinnamils **CL1-12**.

1,2,4-oxadiazole derivatives CH1-4

The 1,2,4-oxadiazole derivatives **CH1-4**, were obtained by adopting the conventional amidoxime route starting from the esters **7** and amidoximes **8** (Scheme S3)[12]. All compounds were regio-selectively obtained in *E,E* geometry.

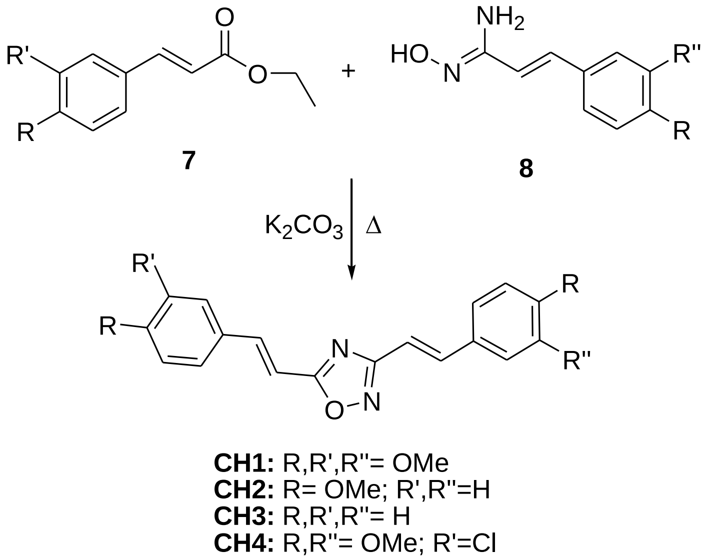

Scheme S3. Synthesis of heterocyclic curcumin-like 1,2,4-oxadiazoles **CH1-4**.

1,3,4-oxadiazole CH5-11.

The 1,3,4-oxadiazole regio-isomers **CH5-11**, , were obtained from the one-pot construction of a diacylhydrazine intermediate, followed by cyclization and starting from the cinnamic acid analogue **4** (Scheme S4)[13]. All compounds were regio-selectively obtained in *E*,*E* geometry.

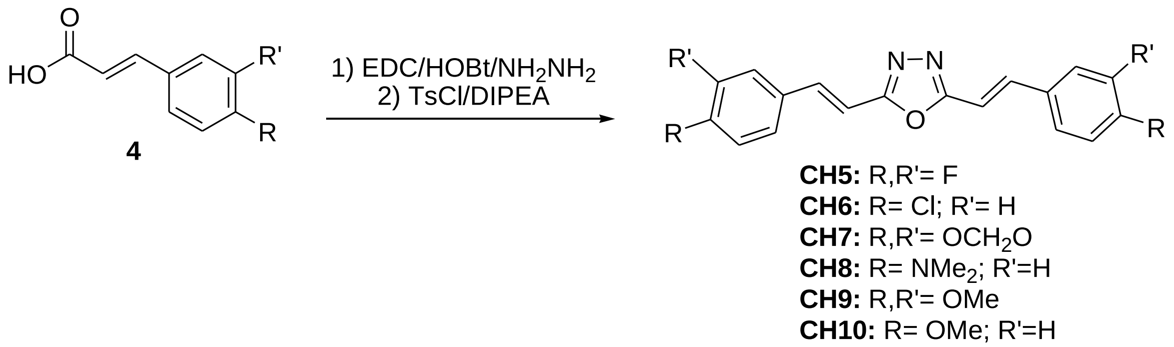

Scheme S4. Synthesis of heterocyclic curcumin-like 1,3,4-oxadiazoles **CH5-11**.

Calebin-A analogs Cal1-9

The synthesis of Calebin-A and its analogs **Cal1-9** was accomplished by coupling, through a nucleophilic substitution reaction, iodo-derivatives **3** and cinnamic acids **4**[14], avoiding the use of protective groups.

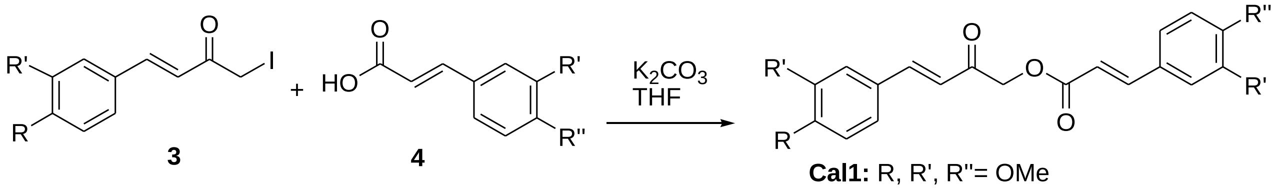

Cal1: R, R', R''= OMe
Cal2: R, R''= OMe; R'= H
Cal3: R, R''= Me; R'= H
Cal4: R = OMOM; R', R''= OMe
Cal5: R, R'= CH_2OCH_2; R'', R'= CH_2OCH_2
Cal6: R, R''= F; R'= H
Cal7: R, R''= Cl; R'= H
Cal8: R, R''= OH; R'= OMe
Cal9: R = F; R', R''= OMe

Scheme S5. Synthesis of Calebin-like compounds **Cal1-9**.

General methods:

General Procedure for Claisen-Schmidt Aldol condensation.

A mixture of aldehyde **1f** (0.1 mol) and acetone (0.11 mol, 8 mL) in 150 mL EtOH/H_2O (1:1) was stirred at room temperature for about 5 min followed by the addition of NaOH (5.2 g, 0.13 mol). The reaction was monitored by TLC. 1 M HCl was used to neutralize the system before extraction three times with EtOAc. The combined organic layer was dried with Na_2SO_4 and then concentrated in vacuo gave the crude product, which was employed without further purification.

(E)-4-(4-fluoro-3-methoxyphenyl)but-3-en-2-one 2f: Yield: 43%. m.p.= 46-49°C. ^{1}H-NMR (250 MHz, $CDCl_3$) δ: 2.39 (s, 3H, CH_3), 3.94 (s, 3H, OCH_3), 6.64 (d, 1H, *J*= 16 Hz, =CH-) 7.09-7.16 (m, 3H, Ar), 7.46 (d, *J*= 16 Hz, =CH-).

***(E)*- 4-(3,4-dimethoxyphenyl)but-3-en-2-one 2j:** Yield: 83%. m.p.= 85-87°C. ^{1}H-NMR (250MHz, $CDCl_3$) δ: 2.38 (s, 3H, CH_3), 3.92 (s, 3H, OCH_3), 3.93 (s, 3H, OCH_3), 6.62 (d, 1H, *J*= 13.5 Hz, =CH) 6.89 (d, 1H, *J*= 6.75 Hz, Ar), 7.08 (d, 1H, *J*= 1.75 Hz, Ar), 7.14 (dd, 1H, *J*= 6.75 Hz, *J*= 1.75 Hz, Ar), 7.48 (d, 1H, *J*= 13.50 Hz, =CH). The ^{1} H NMR spectrum is comparable to that reported from lit[15].

General Procedure for Iodo Ketones 3

Finely powdered CuO (79 mg, 1.0 mmol) and I_2 (254 mg, 1.0 mmol) were added to a well-stirred soln of α,β-unsaturated ketone **2** (1.0 mmol) in *i*-PrOH (5 mL). The mixture was stirred for 5 min and then was heated at 65 °C until disappearance of the reactant (TLC), the mixture was filtered and the solvent was removed under reduced pressure. Purification of the residue by column chromatography yield the target products.

(*E*)-4-(4-fluorophenyl)-1-iodobut-3-en-2-one 3d: Yield: 58%. m.p.= 69-71°C. ^{1}H-NMR (300 MHz, $CDCl_3$) δ: 4.02 (s, 2H, COCH_2I), 6.84 (d, 1H, *J*= 16Hz, =C*H*-), 7.10-7.16 (m, 2H, Ar), 7.59-7.62 (m, 2H, Ar), 7.68 (d, 1H, *J* = 16Hz, =C*H*-). ^{13}C-NMR (75 MHz, $CDCl_3$) δ: 4.9, 116.27 (d, J_{C-F} = 16Hz), 121.9, 128.9 (d, J_{C-F} = 8 Hz), 130.2 (d, J_{C-F} = 3 Hz), 130.6 (d, J_{C-F} = 9 Hz), 143.8, 164.3 (d, J_{C-F} = 251 Hz), 192.0. Anal. Calcd. for $C_{10}H_8FIO$: C, 41.41; H, 2.78; found: C, 41.35; H, 2.85.

(*E*)-4-(4-fluoro-3-methoxyphenyl)-1-iodobut-3-en-2-one 3f: Yield: 83%. m.p.= 70-72°C. ^{1}H-NMR (300 MHz, $CDCl_3$) δ: 3.95 (s, 3H, OCH_3), 4.02 (s, 2H, COCH_2I), 6.79 (d, 1H, *J*= 16Hz, =C*H*-), 7.08-7.17 (m, 3H, Ar), 7.63 (d, 1H, *J*= 16 Hz, =C*H*-). ^{13}C-NMR (75 MHz, $CDCl_3$) δ: 5.0, 113.6, 117.3 (d, J_{C-F} = 19Hz), 122.7, 123.0 (d, J_{C-F} = 7 Hz), 131.4, 144.8, 148.9, 154.9 (d, J_{C-F} = 251 Hz), 192.6. Anal. Calcd. for $C_{11}H_{10}FIO_2$: C, 41.27; H, 3.15; found: C, 41.20; H, 3.30.

General procedure for the Doebner condensation

To a stirred solution of aldehyde **1** (1.00 mmol) in toluene (5 mL) were added malonic acid (156 mg, 1.50 mmol), pyridine (0.12 mL, 1.54 mmol), and aniline (0.01 mL, 0.12 mmol), and the resulting

mixture was refluxed for 18 h. After cooling, the reaction mixture was diluted with AcOEt and 10% HCl aq. and organic phase was separated, dried over Na_2SO_4, and concentrated in vacuo. The residue was chromatographed to give corresponding carboxylic acid **4**. According to this procedure, the following known carboxylic acids were prepared.

Ferulic acid 4a: Yield: 88%. m.p.= 169-172°C. ^{1}H-NMR (DMSO-*d*$_6$, 300 MHz), δ: 3.81 (s, 3H, OC*H*$_3$), 6.36 (d, 1H, *J* = 15.9 Hz, =C*H*-), 7.08 (dd, 1H, *J* = 8.1, 1.4 Hz, Ar), 6.79 (d, 1H, *J* = 8.1 Hz, Ar), 7.28 (d, 1H, *J* = 1.4 Hz, Ar), 7.48 (d, 1H, *J* = 15.9 Hz, =C*H*-), 9.80 (s, 1H, OH), 12.37 (s, 1H, OH)[16].

(*E*)-3-(4-methoxyphenyl)acrylic acid 4g: Yield: 86%. m.p.= 168-169°C. ^{1}H-NMR (DMSO-*d*$_6$, 300 MHz), δ: 3.84 (s, 3H, OC*H*$_3$), 6.43 (d, 1H, *J*= 16.0 Hz, =C*H*-), 7.02 (d, 2H, *J*= 9.0 Hz, Ar), 7.60 (d, 1H, *J*= 16.0 Hz, =C*H*-), 7.69 (d, 2H, *J*= 9.0 Hz, Ar), 12.28 (s, 1H, OH)[16].

(*E*)-3-(4-(dimethylamino)phenyl)acrylic acid 4h: Yield: 89%. m.p.= 214-217°C. ^{1}H-NMR (DMSO-*d*$_6$, 300 MHz), δ: 3.02 (s, 6H, N(C*H*$_3$)$_2$), 6.27 (d, 1H, *J*= 16.0 Hz, =C*H*-), 6.74 (d, 2H, *J*= 10.0 Hz, Ar), 7.49-7.55 (m, 4H, =C*H*-+Ar), 12.01 (s, 1H, OH)[16].

(*E*)-3-(3,4-difluorophenyl)acrylic acid 4i: Yield: 79%. m.p.= 198-199°C. ^{1}H-NMR (DMSO-*d*$_6$, 300 MHz), δ: 6.57 (d, 1H, *J*= 16.0 Hz, =C*H*-), 7.42-7.58 (m, 3H, =C*H*- + Ar), 7.84-7.91 (m, 1H, Ar), 12.49 (s, 1H, OH)[17].

General procedure for the preparation of Cinnamils CL1-12.

Cinnamils **CL** were prepared according to the known literature procedure.[8] In a 50 mL round bottom flask to a solution of diacetyl **6** (2.15 g, 25 mmol) in 10 mL methanol were added aldehyde **1** (100 mmol, 4 equiv), acetic acid (0.03 equiv) and piperidine (0.03 equiv). The reaction mixture was refluxed at 83°C for 3h with stirring. The reaction mixture was cooled to room temperature, solvent was removed and cooled in an ice bath and the precipitate formed was filtered, washed with cold methanol and dried.

(*1E,5E*)-1,6-bis(4-(dimethylamino)phenyl)hexa-1,5-diene-3,4-dione CL4: Yield: 13%. m.p.= 247-248°C. ^{1}H-NMR (250MHz, $CDCl_3$) δ: 3.07 (s, 12H, NC*H*$_3$), 6,70 (d, 4H, *J*= 8.8 Hz, Ar), 7.24 (d, 2H, *J*=16.3 Hz, =C*H*-), 7.56 (d, 4H, *J*= 8.8, Ar), 7.80 (d, 2H, *J*= 16.3 Hz, =C*H*-). ^{13}C-NMR (62.5 MHz, $CDCl_3$) δ: 40.9, 112.6, 116.1, 123.5, 131.8, 148.9, 153.0, 191.1. Anal. Calcd. for $C_{22}H_{24}N_2O_2$: C, 75.83; H, 6.94; N, 8.04; found: C, 75.65; H, 6.85; N, 8.15.

(*1E,5E*)-1,6-bis(3,4-difluorophenyl)hexa-1,5-diene-3,4-dione CL6: Yield: 17%. m.p.= 218-220°C. ^{1}H-NMR (250MHz, $CDCl_3$) δ: 7.22-7.29 (m, 2H, Ar), 7.41-7.55 (m, 6H, =C*H*- + Ar), 7.79 (d, 2H, *J*= 15 Hz, =C*H*-). ^{13}C-NMR (62.5 MHz, $CDCl_3$) δ: 117.8 (d, J_{C-F}= 18 Hz), 118.8 (d, J_{C-F} = 18 Hz), 120.8, 126.5, 131.7, 145.9, 151.0 (d, J_{C-F}= 301 Hz), 153.4 (d, J_{C-F}= 250 Hz), 188.4. Anal. Calcd. for $C_{18}H_{10}F_4O_2$: C, 64.68; H, 3.02; found: C, 64.75; H, 3.15.

(*1E,5E*)-1,6-bis(2-chlorophenyl)hexa-1,5-diene-3,4-dione CL8: Yield: 11%. m.p.= 137-138°C. ^{1}H-NMR (250MHz, $CDCl_3$) δ: 7.27-7.56 (m, 8H, =C*H*- + Ar), 7.79-7.93 (m, 2H, Ar), 8.34 (d, 2H, *J*= 16.3 Hz, =C*H*-). ^{13}C-NMR (62.5 MHz, $CDCl_3$) δ: 122.3, 122.9, 127.9, 128.7, 131.1, 131.3, 132.7, 144.0, 197.0. Anal. Calcd. for $C_{18}H_{12}Cl_2O_2$: C, 65.28; H, 3.65; found: C, 65.45; H, 3.80.

(*1E,5E*)-1,6-bis(3-methoxyphenyl)hexa-1,5-diene-3,4-dione CL9: Yield: 18%. m.p.= 101-102°C. ^{1}H-NMR (250MHz, $CDCl_3$) δ 3.87 (s, 6H, OCH_3), 6.99-7.03 (m, 2H Ar), 7.18 (s, 2H, Ar), 7.24-7.28 (m, 2H, Ar), 7.36 (t, 2H, *J*= 7.7 Hz, Ar), 7.48 (d, 2H, *J*= 15 Hz, =C*H*-), 7.85 (d, 2H, *J*= 15 Hz, =C*H*-); ^{13}C-NMR (62.5 MHz, $CDCl_3$) δ: 56.1, 114.1, 118.3, 120.6, 122.5, 130.7, 136.5, 148.5, 160.7, 189.7. Anal. Calcd. for $C_{20}H_{18}O_4$: C, 74.52; H, 5.63; found: C, 74.60; H, 5.75.

(*1E,5E*)-1,6-bis(3-methoxy-4-(methoxymethoxy)phenyl)hexa-1,5-diene-3,4-dione CL10: Yield: 12%. m.p.= 165-166°C. ^{1}H-NMR (250MHz, $CDCl_3$) δ: 3.53 (s, 6H, OCH_3), 3.96 (s, 6H, OCH_3), 5.31 (s, 4H, OCH_2O), 7.20-7.25 (m, 6H, Ar), 7.39 (d, 2H, *J*= 16.0 Hz, =C*H*-), 7.83 (d, 1H, *J*= 16.0 Hz, =C*H*-). ^{13}C-NMR (62.5 MHz, $CDCl_3$) δ: 56.0, 56.4, 95.1, 110.6, 115.6, 117.9, 124.0, 128.8, 147.7, 149.7, 149.8, 189.1. Anal. Calcd. for $C_{24}H_{26}O_8$: C, 65.15; H, 5.92; found: C, 65.20; H, 5.85.

(*1E,5E*)-1,6-di(benzo[d][1,3]dioxol-5-yl)hexa-1,5-diene-3,4-dione CL11: Yield: 9%. m.p.= 255-256°C. ^{1}H-NMR (250MHz, $CDCl_3$) δ: 6.05 (s, 4H, OCH_2O), 6.84-6.87 (m, 2H, Ar), 7.13-7.18 (m, 4H, *Ar*), 7.31 (d, 2H, *J*= 16.5 Hz, =C*H*-), 7.78 (d, 2H, *J*= 16.5 Hz, =C*H*-)[18].

4,4'-((*1E,5E*)-3,4-dioxohexa-1,5-diene-1,6-diyl)dibenzoic acid CL12: Yield: 14%. m.p.: 236°C (dec.). ^{1}H-NMR (250MHz, DMSO-d_6) δ 7.59 (d, 2H, *J*= 17.5 Hz, =CH-), 7.90 (d, 2H, *J*= 17.5 Hz, =CH-), 7.99-8.21 (m, 8H Ar). ^{13}CNMR (62.5 MHz, DMSO-d_6), δ 122.8, 129.3, 130.0, 133.0, 138.2, 145.8, 166.9, 189.9. Anal. Calcd. for $C_{20}H_{14}O_6$: C, 68.57; H, 4.03; found: C, 68.65; H, 4.90.

Preparation of 1,2,4-oxadiazole 4.

Amidoxime **8** (1 mmol), ethyl ester **7** (1.5 mmol) and K_2CO_3 (3 mmol) were mixed in a glass tube under solvent free conditions and heated at 110°C until complete fusion. The reaction was monitored until completion via TLC. The crude mixture was treated with water (50 mL) and extracted with ethyl acetate (100 mL). The organic layer was dried over anhydrous Na_2SO_4, filtered, concentrated under reduced pressure, and purifed using column chromatography.

3,5-bis(3,4-dimethoxystyryl)-1,2,4-oxadiazole CH1: Yield: 76%. m.p.= 123-125°C. ^{1}H-NMR (250MHz, $CDCl_3$) δ 3.94 (s, 3H, OCH_3), 3.96 (s, 9H, OCH_3), 6.87-6.99 (m, 3H, =C*H*- + Ar), 7.14-7.22 (m, 5H, Ar), 7.68 (d, 1H, *J*= 16.2 Hz, =C*H*-), 7.80 (d, 1H, *J*= 16.2 Hz, =C*H*-); ^{13}C-NMR (62.5 MHz, $CDCl_3$) δ: 55.9 (x2), 56.0 (x2), 107.9, 109.2, 109.4, 110.9, 111.1, 111.2, 121.5, 122.6, 127.4, 128.5, 138.7, 142.6, 149.2, 149.4, 150.4, 151.4, 168.3, 174.7. Anal. Calcd. for $C_{22}H_{22}N_2O_5$: C, 66.99; H, 5.62; N, 7.10; found: C, 67.10; H, 5.55; N, 6.95.

3,5-bis(4-methoxystyryl)-1,2,4-oxadiazole CH2: Yield: 85%. m.p.= 163-165°C. ^{1}H-NMR (250MHz, $CDCl_3$) δ 3.85 (s, 3H, OCH_3), 3.87 (s, 3H, OCH_3), 6.87-6.99 (m, 6H, =C*H*- + Ar), 7.52-7.58 (m, 4H, Ar), 7.68 (d, 1H, *J*= 16.2 Hz, =C*H*-), 7.81 (d, 1H, *J*= 16.5 Hz, =C*H*-); Anal. Calcd. for $C_{20}H_{18}N_2O_3$: C, 71.84; H, 5.43; N, 8.38; found: C, 71.90; H, 5.45; N, 8.55.

3,5-distyryl-1,2,4-oxadiazole CH3: Yield: 82%. m.p.= 113-115°C. ^{1}H-NMR (250MHz, $CDCl_3$) δ 7.05 (d, 1H, *J*= 16.5 Hz, =C*H*-), 7.11 (d, 1H, *J*= 16.2 Hz, =C*H*-), 7.38-7.47 (m, 6H, Ar), 7.60-7.64 (m, 4H, Ar), 7.77 (d, 1H, *J*= 16.2 Hz, =C*H*-), 7.89 (d, 1H, *J*= 16.5 Hz, =C*H*-)[19].

General method of preparation of 1,3,4-oxadiazole

To a solution of cinnamic acid **4** (1.2 mmol) in acetonitrile (7 mL), HOBt (1.2 mmol) and EDC (1.2 mmol) were added. The resulting mixture was stirred for 2 h. Hydrazine hydrate (2 mmol) was successively added and was stirred again for 1 h. The resulting mixture was poured into 10% NaOH aq and extracted with ethyl acetate. The organic layer was dried over sodium sulfate, and concentrated for the next step. To a solution of **4** (1.05 mmol) in acetonitrile (10 mL), was added 1-hydroxybenzotriazole hydrate (1.05 mmol), diisopropylethylamine (1.05 mmol) and EDC (1.05 mmol) and the resulting mixture was stirred for 1.5 h. Previously obtained Hydrazide was added and was stirred again until disappearance of the starting products. The resulting mixture was poured into 10% NaOH aq and extracted with ethyl acetate. The combined organic layers dried over sodium sulfate and concentrated for the obtainment of diacylhydrazide. Diacylhydrazide in acetonitrile (15 mL) was stirred for 1 h with 4-toluensulfonyl chloride (3 mmol) and diisopropylethylamine (2 mmol). At the end of the reaction the crude mixture was concentrated *in vacuo* end treated with water and NaOH. The aqueous phase was extracted with dichloromethane, dried over sodium sulfate and concentrated *in vacuo* The resulting crude was purified by chromatography.

2,5-bis(3,4-difluorostyryl)-1,3,4-oxadiazole CH5: Yield: 67%. m.p.= 233-235°C. ^{1}H-NMR (300MHz, $CDCl_3$) δ: 6.98 (d, 2H, *J*= 16.2 Hz, =C*H*-), 7.19-7.43 (m, 6H, Ar), 7.53 (d, 2H, *J*= 16.2 Hz, =C*H*-). ^{13}C-NMR (62.5 MHz, $CDCl_3$) δ: 110.7, 115.8 (d, J_{C-F}= 17.7 Hz), 117.9 (d, J_{C-F} = 17.7 Hz), 123.9, 131.7, 136.7, 150.8 (d, J_{C-F}= 301 Hz), 151.1 (d, J_{C-F}= 258 Hz), 163.2. Anal. Calcd. for $C_{18}H_{10}F_4N_2O$: C, 62.43; H, 2.91; N, 8.09; found: C, 62.55; H, 2.80; N, 8.15.

2,5-bis(4-chlorostyryl)-1,3,4-oxadiazole CH6: Yield: 82%. m.p.= 229-230°C. ^{1}H-NMR (300MHz, $CDCl_3$) δ: 7.03 (d, 2H, *J*= 16.2 Hz, =C*H*-), 7.40 (d, 4H, *J*= 7 Hz, Ar), 7.51 (d, 4H, *J*= 7 Hz, Ar), 7.56 (d, 2H, *J*= 16.2 Hz, =C*H*-).[20]

2,5-bis((*E*)-2-(benzo[d][1,3]dioxol-5-yl)vinyl)-1,3,4-oxadiazole CH7: Yield: 74%. m.p.= 222-224°C. ^{1}H-NMR (250MHz, $CDCl_3$) δ: 6.04 (s, 4H, OCH_2O), 6.85-6.91 (m, 4H, =C*H*- + Ar), 7.04-7.10 (m, 4H, Ar), 7.50 (d, 2H, *J*= 16.2 Hz, =C*H*-). Anal. Calcd. for $C_{20}H_{14}N_2O_5$: C, 66.30; H, 3.89; N, 7.73; found: C, 66.40; H, 3.90; N, 7.55.

4-((*1E*)-2-(5-(4-(dimethylamino)styryl)-1,3,4-oxadiazol-2-yl)vinyl)-N,N-dimethylbenzenamine CH8: Yield: 59%. m.p.= 217-219°C. ^{1}H-NMR (250MHz, $CDCl_3$) δ: 3.05 (s, 12H, N(C*H*$_3$)$_2$), 6.73-6.87 (m, 6H, =C*H*- + Ar), 7.46-7.54 (m, 6H, =C*H*- + Ar). ^{13}C-NMR (62.5 MHz, $CDCl_3$) δ: 33.4, 104.9, 112.0, 122.9, 128.9, 138.7, 151.4, 164.2. Anal. Calcd. for $C_{22}H_{24}N_4O$: C, 73.31; H, 6.71; N, 15.54; found: C, 73.45; H, 6.60; N, 15.25.

2,5-bis(3,4-dimethoxystyryl)-1,3,4-oxadiazole CH9: Yield: 74%. m.p.= 139-140°C. ^{1}H-NMR (250MHz, $CDCl_3$) δ: 3.94 (s, 6H, OC*H*$_3$), 3.96 (s, 6H, OC*H*$_3$), 6.89-6.97 (m, 4H, =C*H*- + Ar), 7.11-7.17 (m, 4H, Ar), 7.54 (d, 2H, *J*= 16.3 Hz, =C*H*-). Anal. Calcd. for $C_{22}H_{22}N_2O_5$: C, 66.99; H, 5.62; N, 7.10; found: C, 66.90; H, 5.70; N, 7.22[21].

2,5-bis(4-methoxystyryl)-1,3,4-oxadiazole CH10: Yield: 83%. m.p.= 190-192°C. ^{1}H-NMR (250MHz, $CDCl_3$) δ: 3.83 (s, 6H, OC*H*$_3$), 6.85-6.93 (m, 6H, =C*H*- + Ar), 7.48-7.55 (m, 6H, =C*H*- + Ar). Anal. Calcd. for $C_{20}H_{18}N_2O_3$: C, 71.84; H, 5.43; N, 8.38; found: C, 71.75; H, 5.65; N, 8.20.

2,5-bis(3-methoxy-4-(methoxymethoxy)styryl)-1,3,4-oxadiazole CH11: Yield: 67%. m.p.= 128°C (dec.). ^{1}H-NMR (250MHz, $CDCl_3$) δ: 3.67 (s, 6H, OC*H*$_3$), 3.94 (s, 6H, OC*H*$_3$), 5.26 (s, 4H, OC*H*$_2$O), 6.84 (d, 2H, *J*= 16.4 Hz, =C*H*-), 7.13-7.19 (m, 6H, Ar), 7.54 (d, 2H, *J*= 16.4 Hz, =C*H*-). ^{13}CNMR (75 MHz, $CDCl_3$), δ 55.9, 56.4, 95.2, 108.4, 109.6, 115.9, 121.6, 129.2, 138.6, 148.3, 149.9, 163.8. Anal. Calcd. for $C_{24}H_{26}N_2O_7$: C, 63.43; H, 5.77; N, 6.16; found: C, 63.55; H, 5.80; N, 6.00.

General procedure for the preparation of Calebin-A analogs Cal1-9.

Calebin-A and its analogs were prepared by adapting a previously reported procedure[14]. To a solution of the iodo-ketone **3** (1 mmol) in THF (10 mL), cinnamic acid **4** (1.1 mmol) and K_2CO_3 (2 mmol) were added. The mixture was refluxed for 2 h. After cooling to room temperature, the solvent was evaporated in vacuo and the residue treated with water (20 mL) and then extracted with AcOEt (30 mL). The organic layer was dried with Na_2SO_4 and then concentrated in vacuo to gave the crude product which was chromatographed giving desired esters **Cal1-9**.

(*2E*)-(*E*)-4-(3,4-dimethoxyphenyl)-2-oxobut-3-enyl 3-(3,4-dimethoxyphenyl)acrylate Cal1: Yield: 41%. m.p.: 163-165°C. ^{1}H-NMR (300MHz, $CDCl_3$) δ 3.93 (s, 6H, OC*H*$_3$), 3.95 (s, 6H, OC*H*$_3$), 5.08 (s, 2H, C*H*$_2$O), 6.47 (d, 1H, *J* = 15.5 Hz, =CH-), 6.71 (d, 1H, *J* = 16.0 Hz, =CH-), 6.87-6.91 (m, 2H, Ar), 7.09-7.26 (m, 4H, Ar), 7.66 (d, 1H, *J* = 15.5 Hz, =CH-), 7.76 (d, 1H, *J* = 16.0 Hz, =CH-). ^{13}CNMR (75 MHz, $CDCl_3$), δ 55.9 (x2), 56.0 (x2), 67.3, 109.6, 109.9, 111.0, 111.1, 112.6, 114.6, 119.6, 123.0, 123.5, 127.0, 127.2, 144.2, 146.1, 149.2, 149.3, 151.3, 166.5, 192.6. Anal. Calcd. for $C_{23}H_{24}O_7$: C, 66.98; H, 5.87; found: C, 66.75; H, 5.70.

(*2E*)-(*E*)-4-(4-methoxyphenyl)-2-oxobut-3-enyl 3-(4-methoxyphenyl)acrylate Cal2: Yield: 46%. m.p.: 144-145°C. ^{1}H-NMR (300MHz, $CDCl_3$) δ 3.86 (s, 6H, OC*H*$_3$), 5.04 (s, 2H, C*H*$_2$O), 6.47 (d, 1H, *J* = 12.0 Hz, =CH-), 6.74 (d, 1H, *J* = 12 Hz, =CH-), 6.92-6.94 (m, 4H, Ar), 7.51-7.55 (m, 4H, Ar),

7.68 (d, 1H, *J* = 12 Hz, =CH-), 7.77 (d, 1H, *J* = 12 Hz, =CH-). Anal. Calcd. for $C_{21}H_{20}O_5$: C, C, 71.58; H, 5.72; found: C, 71.65; H, 5.50[22].

(2*E*)-(*E*)-2-oxo-4-p-tolylbut-3-enyl 3-p-tolylacrylate Cal3: Yield: 57%. m.p.: 174-176°C. ^{1}H-NMR (300MHz, $CDCl_3$) δ 2.41 (s, 6H, C*H₃*), 5.09 (s, 2H, C*H₂*O), 6.56 (d, 1H, *J* = 16.0 Hz, =CH-), 6.82 (d, 1H, *J* = 16.2 Hz, =CH-), 7.22-7.24 (m, 4H, Ar), 7.45-7.52 (m, 4H, Ar), 7.71 (d, 1H, *J* = 16.3 Hz, =CH-), 7.80 (d, 1H, *J* = 16.0 Hz, =CH-). ^{13}CNMR (75 MHz, $CDCl_3$), δ 21.5, 21.6, 67.5, 115.9, 120.6, 128.3, 128.6, 129.7, 129.8, 131.4, 131.5, 141.1, 141.6, 144.3, 146.2, 166.5, 192.8. Anal. Calcd. for $C_{21}H_{20}O_3$: C, 78.73; H, 6.29; found: C, 78.70; H, 6.20.

(2*E*)-(*E*)-4-(3-methoxy-4-(methoxymethoxy)phenyl)-2-oxobut-3-enyl 3-(3,4-dimethoxyphenyl)acrylate Cal4: Yield: 48%. m.p.: 152-154°C. ^{1}H-NMR (300MHz, $CDCl_3$) δ 3.53 (s, 6H, OCH_3), 3.93 (s, 9H, OCH_3), 5.09 (s, 2H, CH_2O), 5.28 (s, 2H, OCH_2O), 6.49 (d, 1H, *J* = 12 Hz, =CH-), 6.72 (d, 1H, *J* = 12 Hz, =CH-), 6.90 (d, 1H, *J* = 6.3 Hz, Ar), 7.09-7.13 (m, 3H, Ar), 7.16-7.20 (m, 2H, Ar), 7.67 (d, 1H, *J* = 12 Hz, =CH-), 7.76 (d, 1H, *J* = 12 Hz, =CH-). Anal. Calcd. for $C_{24}H_{26}O_8$: C, 65.15; H, 5.92; found: C, 65.15; H, 5.92.**(2*E*)-(*E*)-4-(benzo[d][1,3]dioxol-5-yl)-2-oxobut-3-enyl 3-(benzo[d][1,3]dioxol-5-yl)acrylate Cal5:** Yield: 28%. m.p.: 188-191°C. ^{1}H-NMR (300MHz, $CDCl_3$) δ 5.04 (s, 2H, CH_2O), 6.04 (s, 4H, OCH_2O), 6.43 (d, 1H, *J* = 16.0 Hz, =CH-), 6.68 (d, 1H, *J* = 16.0 Hz, =CH-), 6.83-6.87 (m, 2H, Ar), 7.07-7.10 (m, 4H, Ar), 7.64 (d, 1H, *J* = 16.0 Hz, =CH-), 7.73 (d, 1H, *J* = 16.0 Hz, =CH-). ^{13}CNMR (75 MHz, $CDCl_3$), δ 67.5, 101.6, 101.7, 116.6 (x2), 108.6, 108.7, 114.7, 114.8, 119.5, 124.8, 125.6, 128.5, 128.6, 144.0, 145.9, 148.4, 148.5, 150.3, 166.5, 192.7. Anal. Calcd. for $C_{21}H_{14}O_7$: C, 66.67; H, 3.73; found: C, 66.75; H, 3.60.**(2*E*)-(*E*)-4-(4-fluorophenyl)-2-oxobut-3-enyl 3-(4-fluorophenyl)acrylate Cal6:** Yield: 52%. m.p.: 124-126°C. ^{1}H-NMR (300MHz, $CDCl_3$) δ 5.07 (s, 2H, CH_2O), 6.52 (d, 1H, *J* = 12.0 Hz, =CH-), 6.77 (d, 1H, *J* = 12.3 Hz, =CH-), 7.09-7.14 (m, 4H, Ar), 7.54-7.60 (m, 4H, Ar), 7.69 (d, 1H, *J* = 12.3 Hz, =CH-), 7.78 (d, 1H, *J* = 12.0 Hz, =CH-). ^{13}CNMR (75 MHz, $CDCl_3$), δ 67.6, 130.2 (d, *J* = 10 Hz), 116.3 (d, *J* = 10.1 Hz), 116.6, 116.7, 121.1, 130.2 (d, *J* = 8.5 Hz), 130.3 (d, *J* = 3.5 Hz), 130.5 (d, *J* = 9 Hz), 142.9, 144.9, 164.1 (d, *J* = 251 Hz), 164.3 (d, *J* = 250 Hz), 166.1, 192.4. Anal. Calcd. for $C_{19}H_{14}F_2O_3$: C, 69.51; H, 4.30; found: C, 69.55; H, 4.25.**(2*E*)-(*E*)-4-(4-chlorophenyl)-2-oxobut-3-enyl 3-(4-chlorophenyl)acrylate Cal7:** Yield: 46%. m.p.: 179-181°C. ^{1}H-NMR (300MHz, $CDCl_3$) δ 5.07 (s, 2H, CH_2O), 6.56 (d, 1H, *J* = 12.0 Hz, =CH-), 6.80 (d, 1H, *J* = 12.0 Hz, =CH-), 7.38-7.41 (m, 4H, Ar), 7.51-7.53 (m, 4H, Ar), 7.67 (d, 1H, *J* = 12.0 Hz, =CH-), 7.76 (d, 1H, *J* = 12.0 Hz, =CH-). ^{13}CNMR (75 MHz, $CDCl_3$), δ 67.6, 117.4, 121.7, 129.3, 129.4, 129.5, 129.7, 132.5, 132.7, 136.6, 137.0, 142.8, 144.8, 165.9, 192.3. Anal. Calcd. for $C_{19}H_{14}Cl_2O_3$: C, 63.18; H, 3.91; found: C, 63.20; H, 3.90.

(2*E*)-(*E*)-4-(4-hydroxy-3-methoxyphenyl)-2-oxobut-3-enyl 3-(4-hydroxy-3-methoxyphenyl)acrylate (Calebin-A) Cal8: Yield: 64%. m.p.: 147-148°C. ^{1}H-NMR (300MHz, acetone-d_6) δ 3.91 (s, 3H, OCH_3), 3.94 (s, 3H, OCH_3), 5.09 (s, 2H, CH_2O), 6.52 (d, 1H, J = 15.9 Hz, =CH-), 6.84-6.93 (m, 3H, Ar), 7.11-7.27 (m, 2H, Ar), 7.38-7.39 (m, 2H, Ar), 7.64-7.70 (m, 2H, =CH, overlapped signals), 8.22 (s, 1H, OH), 8.28 (s, 1H, OH). ^{13}CNMR (75 MHz, acetone-d_6), δ 55.4, 55.5, 67.1, 110.5, 110.7, 114.2, 115.2, 115.3, 119.5, 123.3, 123.6, 126.5, 126.6, 143.5, 145.6, 147.9, 149.4, 149.7 166.0, 192.1[14].

(2*E*)-(*E*)-4-(4-fluoro-3-methoxyphenyl)-2-oxobut-3-enyl 3-(3,4-dimethoxyphenyl)acrylate Cal9: Yield: 59%. m.p.: 141-143°C. ^{1}H-NMR (300MHz, $CDCl_3$) δ 3.89 (s, 9H, OCH_3), 5.05 (s, 2H, CH_2O), 6.43 (d, 1H, J = 15.9 Hz, =CH-), 6.72 (d, 1H, J = 15.9 Hz, =CH-), 6.84-6.86 (m, 1H, Ar), 7.06-7.13 (m, 5H, Ar), 7.60 (d, 1H, J = 15.9 Hz, =CH-), 7.71 (d, 1H, J = 15.9 Hz, =CH-). ^{13}CNMR (75 MHz, $CDCl_3$), δ 56.5 (x2), 56.9, 67.9, 110.5, 111.7, 113.5, 115.1, 117.2 (d, J = 22 Hz), 122.0, 122.7 (d, J = 8 Hz), 123.5, 127.8, 131.4, 143.7, 146.7, 148.7 (d, J = 13 Hz), 149.9, 152.0, 154.7 (d, J = 301 Hz), 167.0, 193.1. Anal. Calcd. for $C_{22}H_{21}FO_6$: C, 65.99; H, 5.29; found: C, 65.90; H, 5.35.

References:

1 Wang, Z. *et al. An Efficient Method for the Selective Iodination of α,β-Unsaturated Ketones*. Vol. 2008 (2008).

2 Vander Jagt, D. L., Deck, Lorraine M., Abcouwer, Steve F., Bobrovnikova-Marjon, Ekaterina, Weber, Waylon M. . Cancer treatment using curcumin derivatives. United States patent U.S. Patent 20060276536 (2006).

3 Zhu, J., Mao, M., Ji, H.-J., Xu, J.-Y. & Wu, L. Palladium-Catalyzed Cleavage of α-Allenylic Aryl Ether toward Pyrazolemethylene-Substituted Phosphinyl Allenes and Their Transformations via Alkenyl C–P(O) Cleavage. *Organic Letters* **19**, 1946-1949, doi:10.1021/acs.orglett.7b00213 (2017).

4 Mori, H. *et al.* A novel serine racemase inhibitor suppresses neuronal over-activation in vivo. *Bioorganic & medicinal chemistry* **25**, 3736-3745, doi:10.1016/j.bmc.2017.05.011 (2017).

5 Battisti, A. *et al.* Curcumin-like compounds designed to modify amyloid beta peptide aggregation patterns. *RSC Advances* **7**, 31714-31724, doi:10.1039/C7RA05300B (2017).

6 DiBiase, S. A., Lipisko, B. A., Haag, A., Wolak, R. A. & Gokel, G. W. Direct synthesis of .alpha.,.beta.-unsaturated nitriles from acetonitrile and carbonyl compounds: survey, crown effects, and experimental conditions. *The Journal of Organic Chemistry* **44**, 4640-4649, doi:10.1021/jo00393a037 (1979).

7 Khurana, L. *et al.* Optimization of Chemical Functionalities of Indole-2-carboxamides To Improve Allosteric Parameters for the Cannabinoid Receptor 1 (CB1). *Journal of Medicinal Chemistry* **57**, 3040-3052, doi:10.1021/jm5000112 (2014).

8 Sinu, C. R. *et al.* A Cascade Reaction Actuated by Nucleophilic Heterocyclic Carbene Catalyzed Intramolecular Addition of Enals via Homoenolate to α,β-Unsaturated Esters: Efficient Synthesis of Coumarin Derivatives. *Organic Letters* **15**, 68-71, doi:10.1021/ol303091m (2013).

9 List, B., Doehring, A., Hechavarria Fonseca, M. T., Job, A. & Rios Torres, R. A Practical, efficient, and atom economic alternative to the Wittig and Horner–Wadsworth–Emmons reactions for the synthesis of (E)-α,β-unsaturated esters from aldehydes. *Tetrahedron* **62**, 476-482, doi:https://doi.org/10.1016/j.tet.2005.09.081 (2006).

10 Rehse, K. & Brehme, F. New NO donors with antithrombotic and vasodilating activities, Part 26. Amidoximes and their prodrugs. *Archiv der Pharmazie* **331**, 375-379 (1998).

11 Agarwal, A., Srivastava, K., Puri, S. K. & Chauhan, P. M. Synthesis of 2,4,6-trisubstituted pyrimidines as antimalarial agents. *Bioorganic & medicinal chemistry* **13**, 4645-4650, doi:10.1016/j.bmc.2005.04.061 (2005).

12 Mangione, M. R. *et al.* Photo-inhibition of Aβ fibrillation mediated by a newly designed fluorinated oxadiazole. *RSC Advances* **5**, 16540-16548, doi:10.1039/C4RA13556C (2015).

13 Stabile, P. *et al.* Mild and convenient one-pot synthesis of 1,3,4-oxadiazoles. *Tetrahedron Letters* **51**, 4801-4805, doi:https://doi.org/10.1016/j.tetlet.2010.06.139 (2010).

14 Majeed, M., Nagabhushanam, Kalyanam; Majeed, Anju; Thomas, Samuel Manoharan. Synthesis of Calebin-A and its Biologically Active Analogs United States patent Eur. Pat. 2963007 A1 (2016).

15 Dhuru, S. *et al.* Novel diarylheptanoids as inhibitors of TNF-alpha production. *Bioorganic & medicinal chemistry letters* **21**, 3784-3787, doi:10.1016/j.bmcl.2011.04.040 (2011).

16 Suresh, Kumar, D. & Sandhu, J. S. Bismuth(III) Chloride–Mediated, Efficient, Solvent-Free, MWI-Enhanced Doebner Condensation for the Synthesis of (E)-Cinnamic Acids. *Synthetic Communications* **40**, 1915-1919, doi:10.1080/00397910903162833 (2010).

17 Parmeggiani, F., Ahmed, S. T., Weise, N. J. & Turner, N. J. Telescopic one-pot condensation-hydroamination strategy for the synthesis of optically pure L-phenylalanines from benzaldehydes. *Tetrahedron* **72**, 7256-7262, doi:https://doi.org/10.1016/j.tet.2015.12.063 (2016).

18 Schlenk, H. Notiz über die Kondensation von Diacetyl mit substituierten Benzaldehyden und mit Chloral. *Chemische Berichte* **85**, 901-904, doi:10.1002/cber.19520850912 (1952).

19 Beletskii, E. V., Ignatenko, O. A., Kuznetsov, M. A. & Selivanov, S. I. Oxidative addition of N-aminophthalimide to styryl-1,2,4-oxadiazoles. *Russian Journal of Organic Chemistry* **46**, 678-684, doi:10.1134/s1070428010050143 (2010).

20 Manabe, O., Nagakoshi, T. & Hiyama, H. Syntheses of Oxazoles and Oxadiazoles and their Fluorescence Spectra. *Journal of Synthetic Organic Chemistry, Japan* **26**, 355-360, doi:10.5059/yukigoseikyokaishi.26.355 (1968).
21 Inoue, K., Koshitani, Takeshi, Aoki, Yasunori, Miyake, Yasuhito, Mori, Megumi, Saomoto, Hitoshi, Mori, Takashi. Novel Compound Using Ferulic Acid as Raw Material and Organic Electroluminescent Element Using the Novel Compound. JP 2013014525 (2013).
22 DiMauro, T., M. Use of Nitrogen-containing Curcumin Analogs for the Treatment Alzheimer's disease WO/2010/074971 (2010).
